# Frequency-selective thymic programming by early-life cold stress requires interferon regulatory factor 5

**DOI:** 10.3389/fimmu.2026.1851219

**Published:** 2026-07-15

**Authors:** Shengnan Wang, Liyuan Li, Haokun Li, Kuo Qu, Liying Wang, Wei Sun

**Affiliations:** 1 Medical Basic Research Innovation Center of Airway Disease in North China and Department of Molecular Biology, College of Basic Medical Sciences, Jilin University, Changchun, Jilin, China; 2 Department of Immunology, College of Basic Medical Sciences, Jilin University, Changchun, Jilin, China

**Keywords:** early-life cold stress, frequency-selective immune programming, interferon regulatory factor 4/5, leptin signaling, thymic T cell subsets

## Abstract

**Background:**

Early-life environmental stressors can program immune development, but how the frequency of cold stress shapes thymic programming remains unknown.

**Methods:**

Developmentally matched (day 4, poikilothermic, yolk sac or milk dependent) zebrafish larvae and neonatal mice were exposed to two or four cycles of cold stress (termed 2× or 4× stress). Thymic development (zebrafish) and thymic T cell subsets (mice) were assessed, followed by influenza A virus (IAV) challenge in mice. Mechanisms were explored by RT-qPCR, western/dot blotting, and *irf4/5* transgenic or *irf5* knockout zebrafish.

**Results:**

In *Tg(rag2:DsRed)* zebrafish larvae, 2× stress increased the percentage of larvae exhibiting a DsRed^+^ thymus, without significantly altering overall fluorescence area and MFI of the thymus, whereas 4× stress markedly reduced both of the latter parameters. These opposite phenotypic effects correlated with divergent expression patterns of *lepb*, *lepr*, *irf4/5*, and *stat3*. In neonatal mice, 2× stress enhanced thymic CD3 expression on CD4^+^CD8^+^(DP) and CD4^+^ cells and improved acute survival following IAV infection, whereas 4× stress reduced DP proportion and CD3 expression across multiple subsets, increased CD4^+^ cells, and offered no acute protection against IAV infection, yet unexpectedly enhanced long-term survival when infected as adults. 2× and 4× stress induced divergent dynamics of leptin, IRF4/5 and inflammatory factors. Transgenic overexpression of *irf5*, but not *irf4*, reprogrammed the frequency-selective cold stress response in larvae, *irf5* knockout subsequently abrogated these effects on thymic development and altered baseline expression of leptin signaling-related genes.

**Conclusion:**

Early-life cold stress programs thymic immunity in a frequency-selective manner. Zebrafish genetics establish a causal requirement for IRF5, while mouse data show correlative changes in leptin signaling and IRF4/5. These findings highlight stress frequency as a key determinant of early-life immune programming and suggest potential windows for precise immunomodulation.

## Highlights

- Cold stress frequency selectively exerts opposing effects on thymic immune programming.
- Moderate cold improves acute flu survival whereas excessive cold confers adult protection.
- IRF5 is essential for frequency-selective thymic reprogramming via leptin signaling.

## Introduction

The early-life period, including fetal and neonatal stages, is a critical window for immune system development (1, 2). This window involves the structural and functional maturation of immune organs, which are highly sensitive to environmental stimuli (3, 4). According to the Developmental Origins of Health and Disease (DOHaD) concept, early-life stressors can program physiological functions with lasting impacts on immune competence and homeostasis (5–9). Epidemiological and experimental evidence supports this, linking early-life environment to altered disease risk later in life (10–14).

Cold exposure is a prime example of an environmental stressor with profound, yet complex, immunomodulatory effects. It triggers metabolic reprogramming and brown adipose tissue (BAT) activation (15–17), which interacts deeply with immunity. However, outcomes are contrasting, with moderate cold potentially enhancing defense against neuroinflammation while sustained cold or heat stress may increase susceptibility to viral respiratory infections (18–20). The thymus, as the central organ for T cell development, represents a key target for such environmental programming. It generates a diverse, self-tolerant T cell repertoire (21–23) through a finely regulated process (24–29) that is also vulnerable to external disruption (27, 30). Severe thymic impairment leads to immunodeficiency (31–33). How early-life cold stress frequency influences this pivotal developmental process remains largely unknown.

The link between cold stress and thymic development may involve metabolic-immune signaling axes. The adipokine leptin is a strong candidate (34–36). Cold exposure causes significant fluctuations in leptin levels (37). Leptin binds to receptors on thymic stromal cells and T cell precursors, activating pathways like Janus kinase (JAK)-Signal Transducer and Activator of Transcription (STAT)3. This promotes T cell proliferation, survival, and differentiation while regulating energy balance (38–40). Leptin drives naïve T cells toward pro-inflammatory Th1 phenotypes and suppresses regulatory T cell function (41–44). Mice deficient in leptin or its receptor show thymic atrophy and impaired T cell function (30, 37), confirming its essential role.

How are cold-induced leptin signals converted into developmental instructions in the thymus? We hypothesize that interferon regulatory factor (IRF)4 and IRF5 may serve as downstream signal converters. IRF4 is a core regulator of T cell lineage commitment, positive selection, and regulatory T cell development (21, 45–47). It integrates T cell receptor (TCR) signal strength to guide lineage choices. Strong TCR signals induce high IRF4, which upregulates T-helper-inducing POZ/Krüppel-like factor (ThPOK) and suppresses Runt-related transcription factor 3d (Runx3d), promoting CD4^+^ T cell fate (48). T cell-specific IRF4 deficiency leads to reduced CD4^+^ single-positive cells and a lineage switch to CD8^+^ T cells (49, 50). IRF4 also acts as a cofactor for adipocyte thermogenesis, inhibiting adipogenesis and promoting lipolysis (51). IRF5 is involved in pro-inflammatory signaling and cellular metabolism. Its genetic locus is associated with obesity and insulin resistance (52–55). While not expressed in naïve T cells, IRF5 is present in activated T cells (56) and modulates T cell signaling, migration, and differentiation (57). Recent single-cell RNA sequencing data detect IRF5 transcripts in specific thymocyte subsets (58), suggesting a transient role in T cell fate determination. Both IRF4 and IRF5 interact with the JAK-STAT pathway downstream of leptin signaling (59, 60), positioning them as potential transcriptional bridges from metabolic sensing to immune execution. However, their respective roles in mediating frequency-dependent cold stress responses remain to be distinguished.

Addressing this question requires an experimental system that combines genetic tractability with physiological relevance. The zebrafish and mouse models offer complementary advantages for this purpose. The zebrafish larvae are optically transparent and develop rapidly external development, allowing direct visualization of thymic development and unbiased genetic screening (61). In contrast, the neonatal mouse provides a mammalian physiological context in which T cell subset composition, tissue-specific molecular responses, and functional antiviral outcomes can be assessed (62). Together, these two models enable hypothesis generation in zebrafish followed by rigorous testing in mice, providing a robust framework to dissect the roles of IRF4 and IRF5 in frequency-dependent cold stress responses.

A critical consideration for cross-species comparison is the developmental stage at which the models are used. Although adult zebrafish and adult mice exhibit fundamentally different cold stress response mechanisms (cell-autonomous adaptation versus systemic neuro-endocrine-immune axis), this difference is minimized when using immature stages. Multiple studies have demonstrated that neonatal mice during postnatal days 1–6 are almost completely poikilothermic at low ambient temperatures, unable to maintain constant body temperature through their own metabolism (63, 64). Thus, at postnatal day 4, mice are functionally comparable to poikilothermic zebrafish larvae, both being directly influenced by ambient temperature. The selection of 4-day-old mice and 4 dpf zebrafish is therefore based on their comparable developmental stages regarding thermoregulatory capacity. Moreover, the strategy of combining zebrafish and mice to study cold stress mechanisms has been successfully applied in previous high-impact studies, including the identification of the evolutionarily conserved cold receptor GluK2 (65). Collectively, these findings provide strong methodological precedents supporting our cross-species approach.

Based on this background, we propose that the frequency of early-life cold stress programs thymic development and T cell repertoire formation through the leptin signaling axis and its downstream regulators IRF4 and IRF5, and influences both acute and long-term antiviral immunity. Using neonatal mouse and zebrafish larvae models, this study will systematically elucidate how environmental temperature affects early thymic development and T cell composition, providing new theoretical and experimental insights into early-life immune programming and related disease prevention.

## Materials and methods

### Reagents

Anti-mouse Fluorescence-labeled antibodies: CD3ϵ-FITC (553061), CD4-APC (553051), and CD8α-PE (553032) were from BD Biosciences (NJ, USA). RIPA buffer (P0013C) and PMSF (ST507) were from Beyotime (Shanghai, China). BCA protein assay kit (CW0014S) was from CW Biotech (Beijing, China). Protein-free rapid blocking solution was from Servicebio Technology (Wuhan, China). Rabbit anti-mouse IRF4 Polyclonal antibody (11247-2-AP), Rabbit anti-mouse IRF5 Polyclonal antibody (10547-1-AP), Rabbit anti-mouse Leptin Polyclonal antibody (17436-1-AP), Mouse anti-Beta Actin Monoclonal antibody (66009-1-Ig), and Multi-rAb HRP-Goat anti-Rabbit Recombinant Secondary antibody (H+L) (RGAR001) were from Proteintech Group (USA). Goat anti-Mouse IgG-HRP Antibody (abs20001) was from Absin (Shanghai, China). HRP Conjugated HA tag Recombinant Rabbit Monoclonal Antibody (ET1703-12) was from HuaAn Biotechnology (Hangzhou, China). Super ECL Detection Reagent (36208ES60) was from Yeason Biotechnology (Shanghai, China). TransZol (ET101-01) and cDNA Synthesis Kit (AE301-03) was from Transgen Biotech (Beijing, China). All primers used for RT-qPCR were synthesized and purified at Sangon Biotech Company (Shanghai, China) with HPLC-level purity of 98%. Methyl cellulose (S14012) and Methylene Blue trihydrate (S19043) were from Yuanye Bio-Technology (Shanghai, China). Lipofectamine 2000 (11668-027) was from Invitrogen (USA). Ethyl 3-aminobenzoate methanesulfonate salt (A5040) was from Sigma-Aldrich (German).

### Zebrafish and mice

#### Zebrafish

The wild-type AB strain and *Tg(rag2:DsRed)* transgenic zebrafish were provided by the Experimental Animal Center of the Basic Medical College of Jilin University. The *Tg(ef1a:irf4;ef1a:eGFP)*, *Tg(ef1a:irf5;ef1a:eGFP)*, and *irf5* knockout zebrafish lines were constructed by our research group. Briefly, transgenic zebrafish lines were generated by cloning the coding sequences of *Danio rerio irf4* or *irf5* into a Tol2 transposon vector. In this vector, the gene of interest and an enhanced green fluorescent protein (eGFP) reporter are each under the control of an independent *ef1a* promoter. The resulting plasmid was co-injected with Tol2 transposase mRNA into the cytoplasm of one-cell-stage wild-type (AB) embryos. Founders (F0) were raised to adulthood and outcrossed to AB fish to establish stable transgenic lines. Germline transmission and robust *ef1a* promoter activity were confirmed by ubiquitous GFP fluorescence in F1 progeny. Transgenic lines were maintained through incrossing or outcrossing as required. The *irf5* knockout zebrafish line was generated by CRISPR/Cas9 technology. To generate the *irf5* knockout line, gRNA targeting sites and Cas9 mRNA were first designed and synthesized, then co-injected into embryos. F0 generation fish with *irf5* knockout were screened by PCR and Sanger sequencing, followed by identification of F1 progeny via individual fin-clip genotyping. The *irf5* knockout zebrafish line (*irf5^-/-^*) was maintained as heterozygotes and their homozygous mutants were obtained by incrossing heterozygotes and verified by genotyping.

All zebrafish lines were maintained in a 28 °C recirculating aquatic system under a 14-h light/10-h dark cycle. Adult fish were fed brine shrimp twice daily and solid feed once daily, while larvae started receiving paramecia after 5 days post-fertilization (dpf). All zebrafish husbandry procedures strictly followed the standard protocols recommended in *The Zebrafish Book* and complied with the regulations of the Jilin Provincial Zebrafish Genetic Engineering Laboratory. Zebrafish larvae were obtained through manually controlled mating. Briefly, healthy adult zebrafish were placed in breeding tanks at female-to-male ratios of 2:1 or 2:2, with mating timing regulated by separators. Embryos collected after natural spawning were designated as 0 dpf. Fertilized eggs were incubated in E3 medium containing 0.5 mg/L methylene blue at a constant temperature of 28 °C in a light-controlled incubator. At 3 dpf, healthy hatched larvae were selected for subsequent experiments.

#### Mice

Female ICR mice aged 6–8 weeks and male ICR mice aged 8–10 weeks were purchased from Yisi Experimental Animal Technology Co., Ltd. (Changchun, China). Newborn mice (both sexes, mixed litters) were obtained by breeding the purchased female and male mice. For adult infection experiments following neonatal cold stress, female mice were selected from the surviving cohorts at 6 weeks of age to avoid potential sex-specific effects. All mice were housed in a specific pathogen-free (SPF) barrier environment with a room temperature of 22 ± 2 °C, relative humidity of 50 ± 10%, and a 12-h light/dark cycle, with free access to irradiated feed and sterile drinking water. The animal experiments were approved by the Animal Ethics Committee of the Basic Medical College of Jilin University (2025-658). All procedures were conducted in accordance with the National Institutes of Health Guide for the Care and Use of Laboratory Animals (NIH Publication No. 80-23, revised 1996) and the institutional guidelines of Jilin University. This study was also approved by the Research Project Review Committee of the Jilin Provincial Department of Science and Technology.

The selection of 4-day-old neonatal mice and 4 dpf zebrafish larvae was based on their comparable developmental stages regarding thermoregulatory capacity. In mice, postnatal days 1–6 represent a “functional poikilothermic window” during which neonates cannot maintain constant body temperature under cold exposure (63, 64). This physiological state renders them functionally analogous to poikilothermic zebrafish larvae at 4 dpf, both being sensitive to ambient temperature changes. This developmental alignment provides a rationale for cross-species comparison of early-life cold stress effects on immune programming.

### Cold stress animal models

This study established two types of cold stress animal models: one using 4 days post-fertilization (dpf) zebrafish larvae, and the other using 4-day-old neonatal mice.

#### Zebrafish larvae cold stress models

To determine the maximum tolerable cold stress duration at different developmental stages, zebrafish larvae at 4 dpf and 7 dpf were subjected to continuous 4 °C exposure for various time durations, followed by recovery at 28 °C, and survival was monitored. The results showed that at 4 dpf, no mortality was observed after 6 hours of continuous exposure, whereas 12-hour exposure resulted in 100% mortality (**Supplementary Figure 1A**). At 7 dpf, even 1 h of 4 °C exposure caused mortality, and 6 h continuous exposure led to approximately 90% mortality (**Supplementary Figure 1B**). These results confirm that 4 °C is indeed an extreme temperature for feeding-stage larvae (7 dpf), while 4 dpf larvae possess a unique tolerance window that permits sublethal cold stress studies. Based on these findings, we established cold stress models using 4 dpf larvae. Both single-exposure and cold stress models were established using wild-type AB strain and *Tg(rag2:DsRed)* transgenic zebrafish larvae (4 dpf). The single cold stress model was defined as exposure to 4 °C for 6 h. The cyclic cold stress models consisted of alternating 1 h exposures to 4 °C and 1 h recovery periods at 28 °C, repeated for 2 cycles (2×) or 4 cycles (4×). Control groups were maintained at the standard rearing temperature of 28 °C.

For transgenic zebrafish larvae overexpressing Irf4 or Irf5 [*Tg(ef1a:irf4; ef1a:eGFP)* and *Tg(ef1a:irf5; ef1a:eGFP)*], cyclic cold stress models were established using the 2× and 4× protocols. For *irf5* knockout zebrafish (*irf5^-/-^*), the same 2× and 4× cyclic cold stress protocols were applied to assess the necessity of IRF5 in frequency-dependent cold stress responses.

#### Neonatal mouse cold stress models

A cyclic cold stress model was established using 4-day-old neonatal ICR mice (both sexes, mixed litters). The model involved exposing neonatal mice to 4 °C for 1 h followed by 1 h of recovery at 22 ± 2 °C, which was defined as one cycle (1×) of cold stress. The study included 1×, 2×, 3×, and 4× cyclic cold stress, with 2× and 4× serving as the primary stress paradigms. In this study, “frequency” refers operationally to the number of cold stress cycles applied. Each cycle consists of 1 h of cold exposure at 4 °C followed by 1 h of recovery at 22 ± 2 °C. It is important to note that this operational definition inherently couples cycle number with total cold exposure duration (e.g., 2 h for 2× vs. 4 h for 4×) and total experimental time from the first stress to sampling. However, the termination time of the last cold exposure was synchronized across all groups, and sampling was performed at fixed post-stress intervals (1 h or 24 h), ensuring that recovery durations prior to sampling were comparable across groups. All cold stress procedures and sample collections were performed at consistent times of day across experimental groups to minimize potential circadian influences. Control groups consisted of age-matched neonatal mice maintained at the standard rearing temperature of 22 ± 2 °C.

For adult IAV infection experiments, additional cohorts of neonatal mice were subjected to 2× or 4× cyclic cold stress using the same protocols, then raised under standard housing conditions until 6 weeks of age, at which point female mice were selected for intranasal IAV inoculation.

#### Sampling timepoints

Sampling timepoints for all animal models were calculated from the end of the final cold exposure. For zebrafish larvae models, sampling was performed at 1 h and 24 h post-final cold stress. Fluorescence microscopic observation was conducted at 24 h (5 dpf), 48 h (6 dpf) or 72 h (7 dpf) after the final cold stress.

For neonatal mice, sampling schedules varied based on the subsequent analyses. Before sampling, mice were euthanized with carbon dioxide at a flow rate of 3 L/minute (30% of the chamber volume per minute). Sampling for T cell detection in the thymus, peripheral blood, and lymph nodes was performed at 24 h post-final cold stress. Sampling for RT-qPCR analysis of the thymus and adipose tissue, as well as for Western blotting of the thymus, occurred at both 1 h and 24 h post-final cold stress. Sampling for Dot blotting of serum leptin was carried out at 1, 3, 6, 12, and 24 h following the final cold stress.

### Observation of zebrafish thymus development under a fluorescence microscope

*Tg(rag2:DsRed)* transgenic zebrafish larvae emit red fluorescence during thymic development due to Rag2-DsRed expression. Based on the presence or intensity of fluorescence, images were captured at 10× magnification using a fluorescence microscope (EVOS M700, Invitrogen), while observations and scoring were performed under a stereo fluorescence microscope (SZX10, OLYMPUS) at 3.2× magnification. For imaging, larvae were anesthetized by immersion in Tricaine solution (160 mg/L) and immobilized in 4% methylcellulose. The focal plane was adjusted under bright-field illumination to capture bright-field images, after which the mode was switched to fluorescence. Fine adjustments were made to achieve optimal focus before capturing the fluorescence images. Quantitative analysis of the fluorescence area and mean fluorescence intensity (MFI) in the thymic region was performed using ImageJ software. Briefly, images were loaded into ImageJ, and a single channel was extracted (Image > Color > Split Channels). A uniform threshold of 15 to 255 was applied (Image > Adjust > Threshold), and the fluorescent region was selected. Measurement parameters were set using the default algorithm (Analyze > Set Measurements) with the following options selected: Area, Min & max gray value, Integrated Density, Mean Gray Value, and Limit to Threshold. Measurements were then obtained (Analyze > Measure).

For *Tg(ef1a:irf4; ef1a:eGFP)* and *Tg(ef1a:irf5; ef1a:eGFP)* transgenic zebrafish larvae, images were directly captured at 3.2× magnification using the fluorescence microscope (SZX10, OLYMPUS). For Tg(*ef1a:irf5; ef1a:eGFP*) transgenic zebrafish and *irf5* knockout zebrafish (*irf5^-/-^*) crossed with the *Tg(rag2:DsRed)* background, thymic development was assessed using the same fluorescence imaging and ImageJ quantification protocols as described for *Tg(rag2:DsRed)* larvae.

### Mouse experiments on influenza virus infection following neonatal cyclic cold stress

#### Neonatal IAV infection

Four-day-old neonatal ICR mice subjected to 2× or 4× cyclic cold stress received intranasal inoculation with mouse-adapted FM1 H1N1 influenza A virus (IAV) at 24 h after the final cold exposure. Age-matched control mice maintained at 22 ± 2 °C were similarly infected with FM1 IAV via intranasal instillation. Following infection, body weight was measured daily until mortality occurred. Mortality was recorded and survival duration was calculated for each group, with 12 mice per group. On day 3 post-FM1 IAV infection, 6 mice from each group were euthanized with carbon dioxide at a flow rate of 3 L/minute (30% of the chamber volume per minute) for gross observation and pathological examination of lungs, while the remaining 6 mice were retained for continued survival observation.

In a separate cohort of mice subjected to the same cold stress and infection protocols, euthanasia was performed on day 7 post-infection to observe T cell recruitment in the lungs. The trachea and lungs were then harvested, and bronchoalveolar lavage (BAL) was conducted by slowly injecting 1 mL per mouse of PBS containing 2% fetal bovine serum (FBS) into the trachea using a 1 mL syringe. The collected BAL cells were subsequently used for flow cytometric analysis.

#### Adult IAV infection

To investigate whether neonatal cyclic cold stress influences anti-viral immunity in adulthood, additional cohorts of four-day-old neonatal ICR mice were subjected to 2× or 4× cyclic cold stress or maintained at 22 ± 2 °C (control). Body weight was measured dynamically from the neonatal period to adulthood. At 6 weeks of age, all mice were intranasally inoculated with the mouse-adapted FM1 H1N1 IAV. Post-infection, body weight and survival were monitored daily. Survival rates were calculated for each group.

### Reverse transcription & quantitative polymerase chain reaction

All samples for RT-qPCR analysis, including whole zebrafish larvae, neonatal mouse adipose and thymus tissues were processed using Trizol reagent for total RNA extraction. mRNA was then reverse transcribed into cDNA using a cDNA Synthesis Kit. Quantitative PCR was performed on the StepOne™ Real-Time PCR System (Applied Biosystems, Foster City, CA, USA) using a three-step SYBR Green fluorescence method with gene-specific primers (**Table 1**) to amplify target genes. The reaction conditions were as follows: 94 °C for 5 s (1 cycle); followed by 40 cycles of 94 °C for 5 s, 60 °C for 30 s, and 72 °C for 15 s. Target mRNA expression levels were normalized to *β-actin* mRNA and analyzed using the 2^–ΔCt^ and 2^–ΔΔCt^ method. Each qPCR plate included a no-template control.

**Table 1:** qPCR primers for target gene amplification from zebrafish and mouse.

Species	Genes	Forward primer (5’-3’)	Reverse primer (5’-3’)
	*actb*	CCTCTCTTGCTCCTTCCACC	TACTCCTGCTTGCTGATCCAC
	*rag2*	TCAACCTCTGGCTCACTCACT	TCCCAGTTGGACATGAGCGT
	*lepb*	AATATCATCCCTGGTGGCCGT	CAATGGGGTTGTCGATGTCAG
	*lepr*	AGGTCGTTCATGGGTTGCTC	CCTCTGACTCCTGCGTTGTG
Zebrafish	*irf4*	ACACAGCACCCTGCGATAA	TGCTGGTGGTCAGTTCTTTCA
	*irf5*	GCACAGATCAACAGCGGGAA	TTCCTGGTACTTGCCGGTTTC
	*stat3*	CATTCGAGGTTCACGCAAGT	TCACTATTGGTTCGGCCTCC
	*jak1*	AGAAGACCACCGAGGGAACA	TTCCCAAGCACTCGTTCTCA
	*jak2*	CATCCTGGTGGAGAGCGAGT	ACTCCGAAACTCCAGACGTC
	*Actb*	GATCAAGATCATTGCTCCTCCTG	AGGGTGTAAAACGCAGCTCA
	*Lep*	AGCAGTGCCTATCCAGAAAGT	CCCAGGAATGAAGTCCAAGC
Mice	*Lepr*	ACCTGAAAGCCACCAGACCT	GTGATTGGATTGTGCTGGGTG
	*Irf4*	CTTTGAGGAATTGGTCGAGAGG	GAGAGCCATAAGGTGCTGTCA
	*Irf5*	GGTCAACGGGGAAAAGAAACT	CATCCACCCCTTCAGTGTACT
	*Stat3*	GAAAGTGTCCTACAAGGGCGA	ACCAGCAACCTGACTTTCGTG

### Flow cytometry

Single-cell suspensions from mouse thymus or lymph nodes were prepared by mechanical grinding and filtration through a 300-mesh sieve. Alternatively, peripheral blood mononuclear cells were isolated after red blood cell lysis. These cell preparations were used for flow cytometric analysis of T cells and their subsets. For cell surface staining, samples containing 1×10^6^ cells were incubated with fluorescently labeled antibodies, including anti-CD3ϵ-FITC, anti-CD4-APC, and anti-CD8α-PE, on ice for 30 min in dark. Following staining, all samples were washed with PBS and analyzed using an BD Accuri C6 flow cytometer (BD Biosciences, NJ, USA).

A sequential gating strategy was employed to identify thymocyte subsets. First, cells were plotted on a forward scatter area (FSC-A) versus side scatter area (SSC-A) dot plot. The main population of events with characteristic lymphocyte light-scatter properties was gated as P1 to exclude debris. From cells in the P1 gate, thymocytes were directly visualized on a CD4 versus CD8 dot plot to distinguish CD4^-^CD8^-^ double-negative (DN), CD4^+^CD8^+^ double-positive (DP), CD4^+^ single-positive (SP), and CD8^+^ SP subsets and CD3 mean fluorescence intensity (MFI). BAL cells were analyzed for CD3ϵ expression. The CD3ϵ^+^ population was then further visualized on a CD4 versus CD8 dot plot to distinguish and quantify the DN, DP, CD4 SP and CD8 SP subsets.

### Histopathology and pathological scoring

Lung tissues obtained from influenza virus-infected mice were fixed in 10% formalin, followed by paraffin embedding and sectioning. The paraffin sections were deparaffinized and rehydrated, then subjected to hematoxylin and eosin (H&E) staining. Briefly, sections were first stained with hematoxylin, differentiated with 1% acid alcohol, and blued under running water. This was followed by counterstaining with eosin, dehydration, clearing with xylene, and mounting with neutral balsam. Finally, sections were scanned and imaged using a Leica M8 digital slide scanning microscopy system.

The severity of lung tissue lesions was scored based on four parameters: alveolar septal thickening, hemorrhage, inflammatory cell infiltration, and focal lymphocyte aggregation. Compared with normal mouse lung tissue sections, the scoring criteria were as follows: 0 points for no lesions, 1 point for lesions involving <25% of the tissue, 2 points for 25–50%, 3 points for 50–75%, and 4 points for >75% involvement. The total scores (sum of the four parameters) was taken as the final lesion score for each lung tissue.

### Western/dot blotting

For Western blotting, cold RIPA buffer containing 1 mM PMSF was added to homogenates of mouse thymus tissue or cell samples to lyse the cells. The lysates were then sonicated three times using an ultrasonic cell disruptor, with each cycle consisting of 3 s of sonication followed by a 5 s interval. Total protein concentration was determined using a BCA protein assay kit (CW0014S, CW Biotech, Beijing, China). Target proteins were separated by 12% SDS-PAGE and subsequently transferred onto a PVDF membrane (Millipore, Billerica, MA, USA). Prior to immunoblotting, the membrane was trimmed according to the molecular weight positions indicated by the protein marker. The trimmed membrane was blocked in Protein-free Rapid Blocking Solution (Servicebio Technology, Wuhan, China) for 20 min, followed by incubation with respective primary antibodies such as anti-IRF4, anti-IRF5, anti-β-actin or anti-HA in separate hybridization bags at 4 °C overnight. The following day, for the membrane of anti-IRF4, anti-IRF5, and anti-β-actin were washed three times with PBST (PBS/0.05% Tween 20), incubated with HRP-conjugated secondary antibody at room temperature for 1 h. Finally, all membranes were washed three times with PBS.

For dot blotting, serum samples obtained from mouse peripheral blood were directly spotted onto a nitrocellulose (NC) membrane (Schleicher & Schuell), with 1 μL applied per sample. The membrane was air-dried, transferred into a hybridization bag, and incubated with Protein-free Rapid Blocking Solution for 20 min. Subsequently, the membrane was incubated with anti-Leptin antibody at room temperature for 2 h, followed by three washes with PBS. An HRP-conjugated secondary antibody was then added and incubated at room temperature for 1 h, after which the membrane was washed three times with PBS.

All membranes processed with primary and secondary antibodies were incubated with Super ECL Detection Reagent and imaged using a WEALTEC imaging system. The resulting bands or dots were quantitatively analyzed for gray values using ImageJ software. Each membrane included samples from the same control group to standardize comparisons across different membranes. The final results were expressed as the ratio of the normalized sample gray value to that of the internal control protein.

### Statistical analysis

Data are shown as mean ± SD. All calculations and statistical analysis were performed using GraphPad Prism 10.1.2 (GraphPad Software, USA) for Windows. Comparisons between groups were conducted using unpaired t-tests. *p* < 0.05 was regarded as statistically significant. All experiments were performed at least three independent times.

## Results

### Effects of cold stress on thymic development and metabolic-immune gene expression in zebrafish larvae

**Figure 1:**
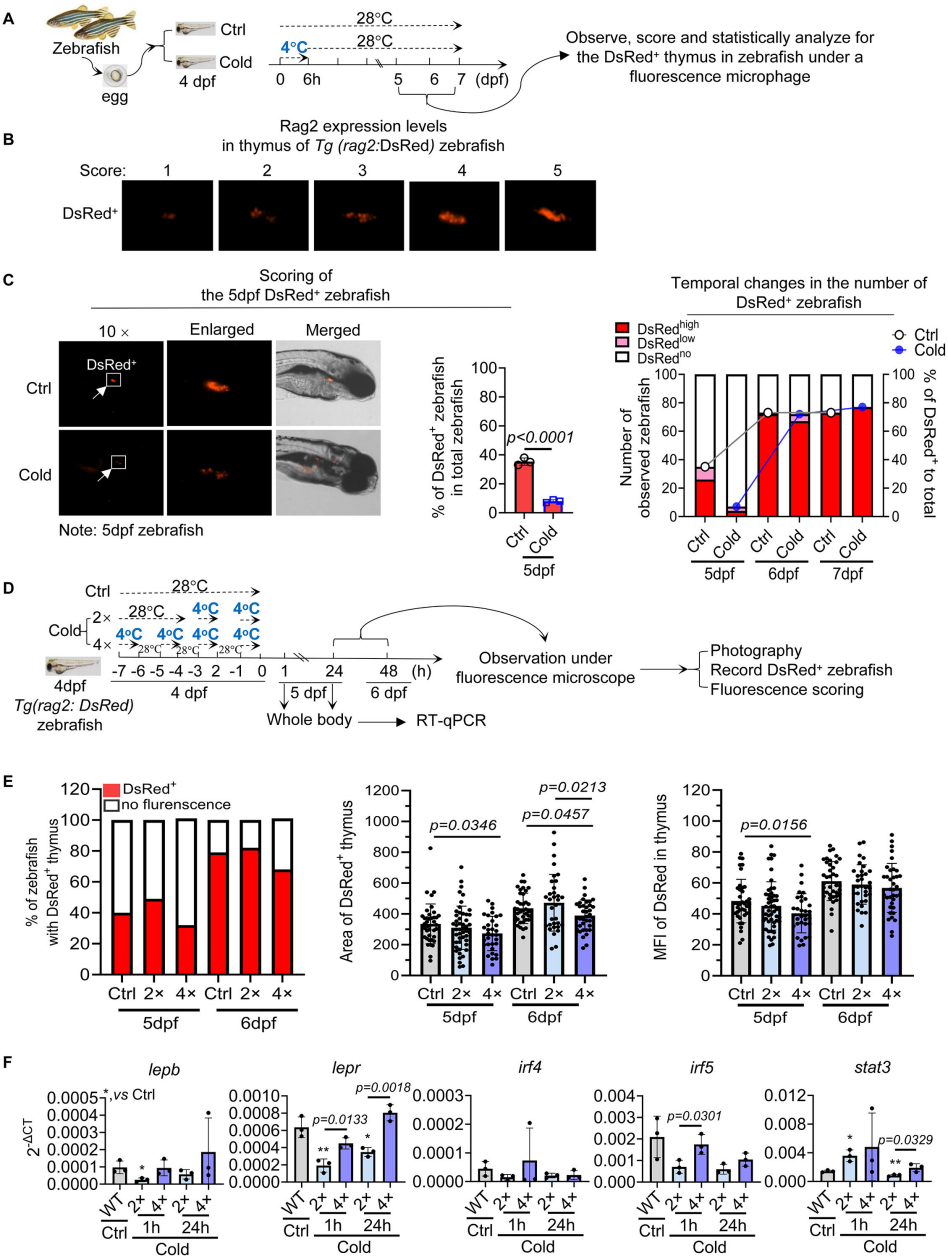
Frequency-selective effects of cyclic cold stress on thymic development and leptin-IRF/Stat3 signaling in zebrafish larvae. **(A)** Experimental design. *Tg(rag2:DsRed)* zebrafish larvae at 4 days post-fertilization (dpf) were subjected to a single 6 h cold stress at 4 °C followed by assessing thymic development at 24 h (5 dpf), 48 h (6 dpf), and 72 h (7 dpf) post-stress. **(B)** Representative fluorescence images of DsRed^+^ thymus at indicated time points. **(C)** Quantification of thymic development after a single 6 h cold stress. **(D)** Experimental procedure for cyclic cold stress (2× or 4× cycles of 1 h at 4 °C followed by 1 h at 28 °C per cycle). **(E)** Quantification of thymic fluorescence area and mean fluorescence intensity (MFI) after cyclic cold stress. **(F)** Relative mRNA expression levels of *lepb*, *lepr*, *irf4*, *irf5* and *stat3* at 1h and 24 h post-stress, as determined by RT-qPCR. ^*^p < 0.05, ^**^p < 0.01. Each data point represents a sample pooled from 10 zebrafish.

To investigate the impact of cold stress on early-life thymic development, we first performed pilot experiments to find the cold tolerance window of zebrafish larvae. As shown in **Supplementary Figures 1A**, **4** dpf larvae withstood continuous 4 °C exposure for up to 6 h and remained alive. Comparatively, 7 dpf larvae were markedly more sensitive with mortality observed after 1 h of continuous cold exposure and an approximate 90% death after 6 h of cold exposure. (**Supplementary Figure 1B**). Therefore, all subsequent cold stress experiments were performed using 4 dpf larvae, with either a single continuous 6 h exposure or a cyclic exposure paradigm (1 h cold/1 h recovery). Accordingly, we first subjected *Tg(rag2:DsRed)* zebrafish larvae (4 dpf) to a single 6 h cold stress at 4 °C and assessed their thymic development at 24 h (5 dpf), 48 h (6 dpf) and 72 h (7 dpf) post-stress (**Figure 1A**). After scoring based on red fluorescence intensity (**Figure 1B**), we found that cold stress delayed thymic development, as evidenced by a significant decrease in the proportion of DsRed^+^ zebrafish at 24 h post-stress (5 dpf). Noticeably, this effect was reversible, as no significant difference was observed at 48 h and 72 h (**Figure 1C**). To explore the underlying mechanisms, we performed whole-fish RNA sequencing and found that cold stress triggered differential gene expression enriched in energy metabolism and immune-related pathways, particularly the adipocytokine signaling pathway (**Supplementary Figure 2**). Within the leptin signaling axis, cold stress reduced *lepb* and *jak1* mRNA levels. Protein-protein interaction network analysis further revealed that *Lepb/Lepr* indirectly interact with *Irf4* and *Irf5* (**Supplementary Figure 2**). These results indicate that a single cold stress preferentially impacts the leptin-Irf4/5 signaling module. Given that a single prolonged cold exposure represents only an acute stress scenario, we next tested whether a cyclic short-duration cold stress paradigm could produce distinct effects depending on stress frequency. Zebrafish larvae were subjected to two cycles (2×) or four cycles (4×) cycles of cold stress (1× cycle: 1 h at 4 °C followed by 1 h at 28 °C) (**Figure 1D**). At 24 h (5 dpf) and 48 h (6 dpf) post-stress, DsRed^+^ larvae, thymic fluorescent area and mean fluorescence intensity (MFI) were assessed. Compared to control (Ctrl), 2× stress increased DsRed^+^ proportion without altering thymic fluorescent area and MFI; 4× stress reduced both DsRed^+^ proportion and thymic fluorescent area at both time points (and thymic fluorescent area versus 2× stress at 6 dpf), whereas thymic MFI was significantly lower than Ctrl only at 5 dpf, recovering to levels comparable with both Ctrl and 2× groups by 6 dpf (**Figure 1E**). Compare to Ctrl, the 2× stress suppressed mRNA levels of leptin signaling genes (*lepb, lepr, stat3*) and *irf4/5*, except for a transient *stat3* increase at 1 h. In contrast, the 4× stress reversed this effect, significantly elevating *lepr*, *irf5*, and *stat3* transcripts (**Figure 1F**). Collectively, these findings demonstrate that cold stress shapes thymic development and metabolic-immune gene expression in a frequency-selective manner in zebrafish larvae: the 2× and 4× stress produce opposing effects, whereas the 1× stress (single 6 h exposure) only induced reversible delay. The opposing effects of 2× and 4× cyclic cold stress on both thymic phenotype and leptin/IRF/Stat3 signaling provided a critical foundation for our subsequent investigation into whether similar frequency-selective mechanisms operate in the mammalian thymus.

### Cold exposure tolerance in neonatal mice and associated changes in thymic T cell subset proportions

**Figure 2:**
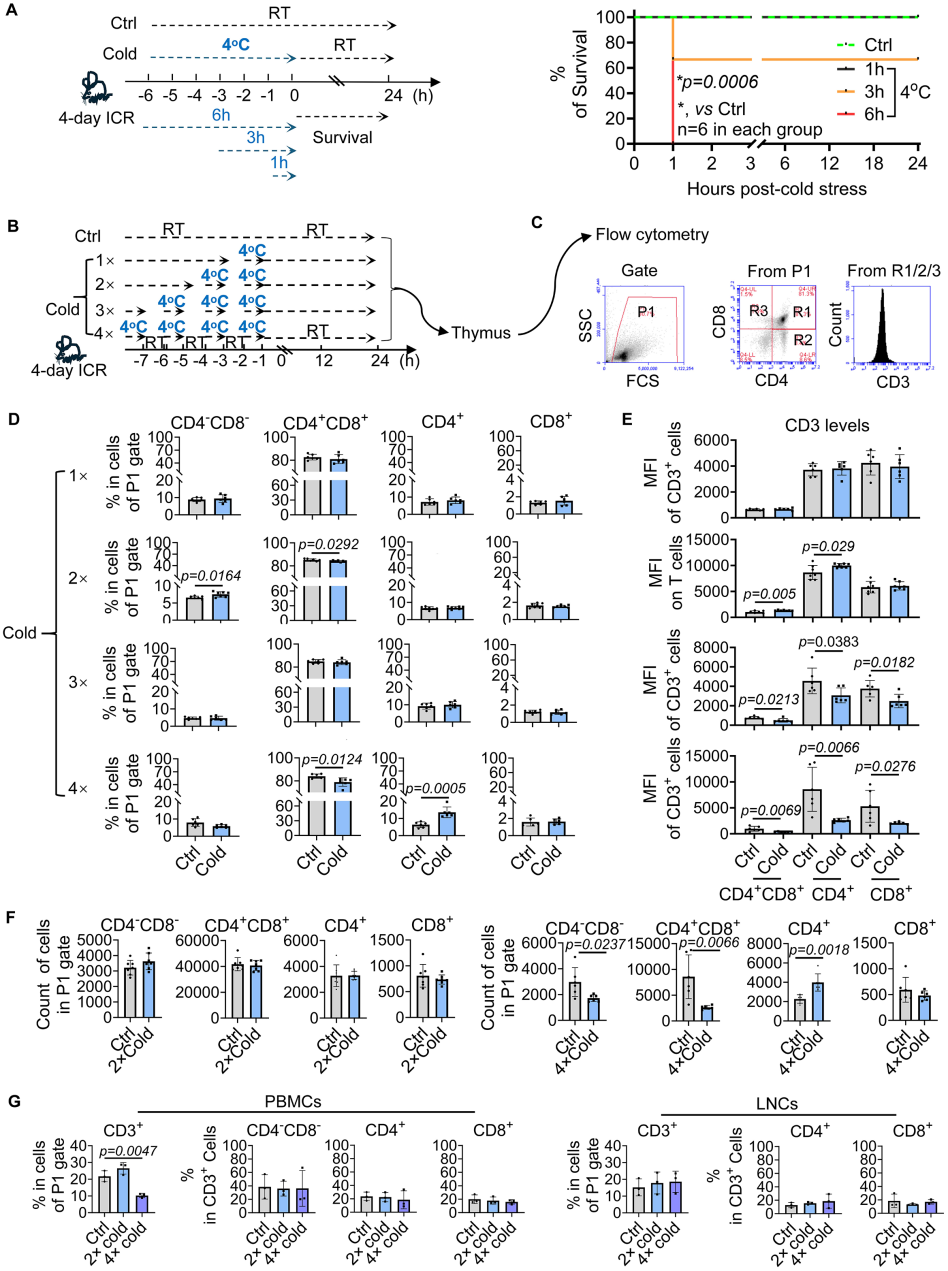
Effects of cyclic cold stress on the T cell developmental landscape in the thymus of neonatal mice. **(A)** Survival of neonatal mice following cold stress exposure of varying durations. **(B)** Schematic diagram of the cyclic cold stress experimental procedure. **(C)** Gating strategy for flow cytometric analysis of mouse thymic T cells. Thymocytes were first gated on FSC-A/SSC-A to exclude debris (P1), then CD4 versus CD8 plots were used to identify DN, DP, CD4 SP, and CD8 SP subsets. DP, CD4 SP, and CD8 SP cells were further analyzed for surface CD3 MFI. **(D)** Proportional changes of thymic DN, DP, CD4 SP, and CD8 SP subsets at 24 h post 1× to 4× cyclic cold stress. **(E)** Surface CD3 MFI analysis on DP, CD4 SP, and CD8 SP subsets. **(F)** Cell counts of DN, DP, CD4 SP, and CD8 SP subsets in the thymus of 2× and 4× stress groups. **(G)** Proportional changes in total T cells and major T cell subsets in the peripheral blood and lymph nodes of mice at 24 h post 2× or 4× cyclic cold stress. Each data point represents one mouse.

Having established that cyclic cold stress induces frequency-selective effects on thymic development and metabolic-immune gene expression in zebrafish as shown in **Figure 1**, we next tested whether a similar paradigm could modulate thymic T cell subset composition in a mammalian model. To test this in neonatal mice, we first needed to determine the maximum tolerable duration of a single cold exposure. Neonatal mice were subjected to a single 4 °C cold stress for 6 h, 3 h or 1 h. All mice died within 1 h after the 6 h exposure; mortality was also observed in the 3 h group, whereas the 1 h exposure caused no mortality (**Figure 2A**). Thus, neonatal mice can tolerate a single 1 h cold stress. We then assessed the effects of a cyclic cold stress paradigm consisting of 1 to 4 cycles of 1 h at 4 °C followed by 1 h at room temperature (termed 1×–4× stress) on thymic T cell subset proportions and their CD3 expression levels (**Figure 2B**). Using our established flow cytometry gating strategy (**Figure 2C**), which analyzed CD4^-^CD8^-^double negative (DN), CD4^+^CD8^+^ double positive (DP), CD4^+^ single positive (CD4 SP), and CD8 SP subsets directly from CD4 versus CD8 plots without prior CD3 gating, we found no significant differences in the proportions of these four thymocyte subsets between Ctrl and mice subjected to 1× or 3× stress. However, significant alterations occurred after 2× and 4× stress. Compared to Ctrl, the 2× stress showed a significantly increased proportion of DN cells and a decreased proportion of DP cells, but these changes were each less than 1%. In contrast, the 4× stress exhibited a reduction of approximately 5% in DP cells and a marked increase of about 7% in CD4 SP cells (**Figure 2D**). We next analyzed the surface CD3 MFI on DP, CD4 SP, and CD8 SP subsets. Relative to Ctrl, the 1× stress again showed no difference; the 2× stress exhibited significantly increased CD3 MFI on both DP and CD4 SP cells; and both the 3× and 4× stress showed significantly reduced CD3 MFI on DP, CD4 SP, and CD8 SP cells (**Figure 2E**). Given that the most pronounced changes were observed in the 2× and 4× stress groups, we focused subsequent cell count analyses on these two groups. Compared to Ctrl, the 2× stress showed no significant differences in counts of the four subsets, whereas the 4× stress displayed significantly reduced DN and DP counts, a significantly increased CD4 SP count, and no change in CD8 SP count (**Figure 2F**). In peripheral blood, the 4× stress exhibited a significant decrease in CD3^+^ T cell proportion, with no changes in other blood T cell subsets or in lymph node T cell populations (**Figure 2G**). This reduction in blood CD3^+^ T cells coincided with the severe loss of their DP precursors in the thymus shown in **Figures 2D, F**. Together, these data indicate that cyclic cold stress regulates thymic T cell subset composition and CD3 expression in a frequency-selective manner in neonatal mice, with the 2× stress producing a unique pro-maturation signature, while the 3× and 4× stress promote CD4 SP differentiation and suppress CD3 expression, with the 4× stress showing more pronounced suppression.

### Differential effects of neonatal cyclic cold stress on anti-influenza virus immunity from acute protection to long term reprogramming

**Figure 3:**
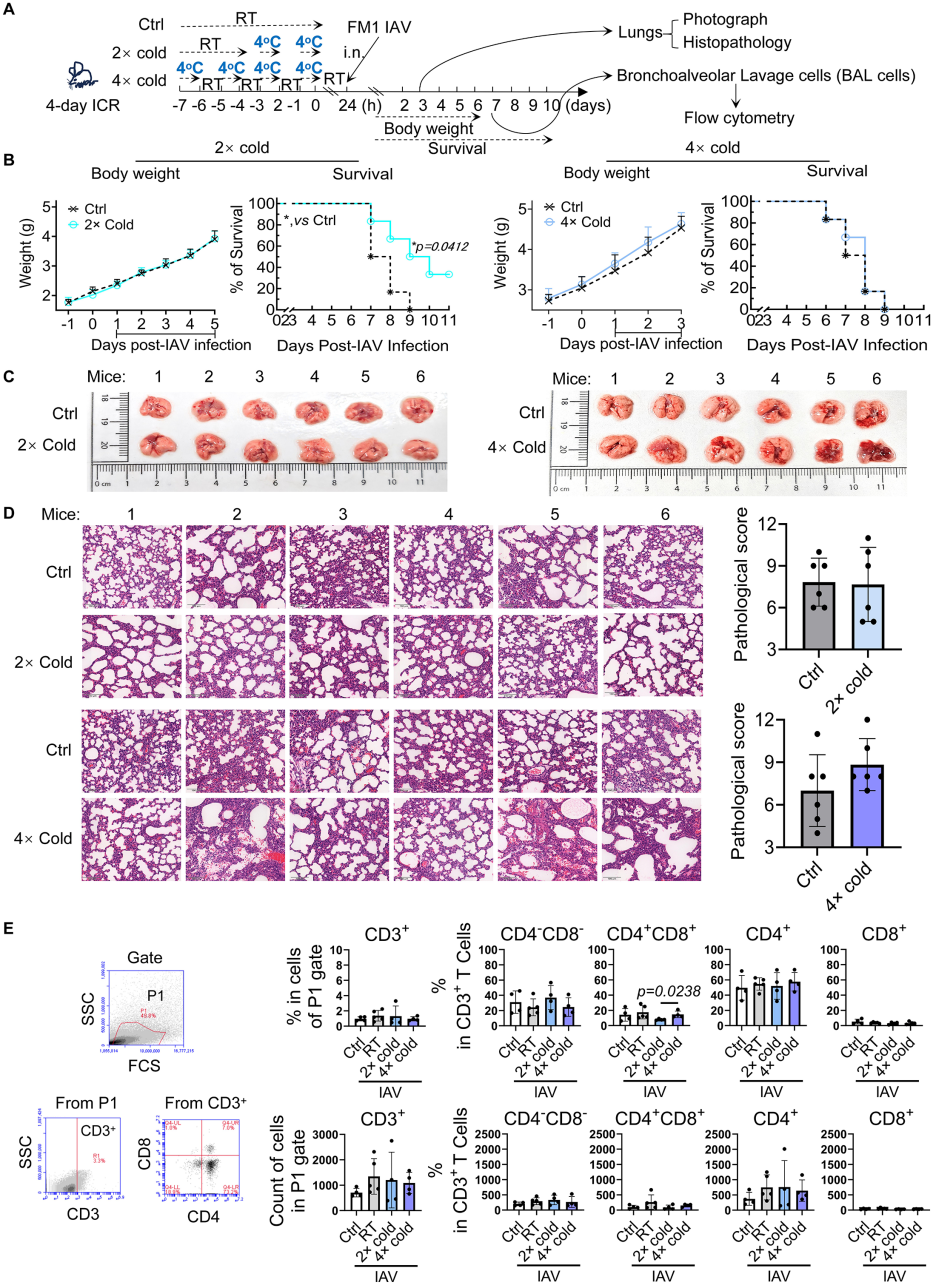
Effects of 2× versus 4× cold stress on influenza virus infection in neonatal mice. **(A)** Experimental timeline of the cold stress model in 4-day-old neonatal mice and subsequent intranasal infection with influenza virus. Following influenza virus infection, mouse body weight and survival were monitored. Body weight was monitored until the first mouse death occurred. Survival was monitored until day 11 post-infection. **(B)** Body weight change and survival curves of mice from different cold stress groups following influenza virus infection. **(C)** Gross photographs of lungs from each group of mice on day 3 post-influenza virus infection. **(D)** Representative H&E-stained lung tissue sections and corresponding lung inflammation pathology scores for each group on day 3 post-influenza virus infection. **(E)** Detection of T cells and their subsets in bronchoalveolar lavage (BAL) cells from mice on day 7 post-influenza virus infection. Neonatal mice subjected to 2× or 4× cold stress for 24 h or kept at room temperature (RT) were intranasally infected with FM1 IAV. On day 7 post-infection, bronchoalveolar lavage was performed, and the percentages and counts of CD3^+^ T cells and their subsets in BAL cells were analyzed by flow cytometry. Given that the proportion of total CD3^+^ T cells in neonatal mouse BAL is less than 3%, the analyzed data are presented for reference only. Age-matched uninfected mice served as controls (Ctrl). Each symbol represents one mouse.

Given the opposing effects of 2× and 4× cyclic cold stress on thymic T cell subsets, we next assessed their impact on anti-viral immunity using a neonatal mouse model of influenza virus infection. Four-day-old neonatal mice were subjected to 2× or 4× stress, and 24 h later, they were intranasally inoculated with the mouse-adapted FM1 strain of influenza A virus (FM1 IAV). Body weight and survival were monitored daily for 11 days, and pulmonary pathology was assessed on day 3 post-infection (**Figure 3A**). No significant differences in body weight changes were observed across groups. However, the 2× stress significantly prolonged survival compared to Ctrl (RT), whereas the 4× stress showed no such improvement (**Figure 3B**). Gross lung examination revealed no differences between the 2× stress and Ctrl, but pulmonary surface hemorrhage was noted in some 4× stress mice (**Figure 3C**). Lung histopathology scores showed no significant differences among groups (**Figure 3D**). These results indicate that the outcome of influenza virus infection is critically determined by the intensity of prior cold stress. To explore local pulmonary immune responses and determine whether T cells begin to infiltrate the alveolar space by day 7 post-infection (approximately 12 days of age), we analyzed T cells in bronchoalveolar lavage (BAL). It has been reported that BAL from 2-day-old neonatal mice contains few, if any, T cells (66). In our study, CD3^+^ T cells comprised less than 3% of total BAL cells at day 7 post-infection. Although this proportion remains low, it suggests that a small number of T cells do begin to appear in the alveolar space by 12 days of age. Within this limited population, no significant differences were observed in major T cell subsets between stress groups and Ctrl, except for a higher percentage of DP T cells in the 4× stress than in the 2× stress (**Figure 3E**). These data indicate that T cell infiltration into the neonatal alveolar space, though minimal, is not absent, and the presence of DP T cells may reflect either ongoing thymic output or local immune responses. Nevertheless, given the overall low T cell abundance, these findings should be interpreted with caution and considered exploratory.

**Figure 4:**
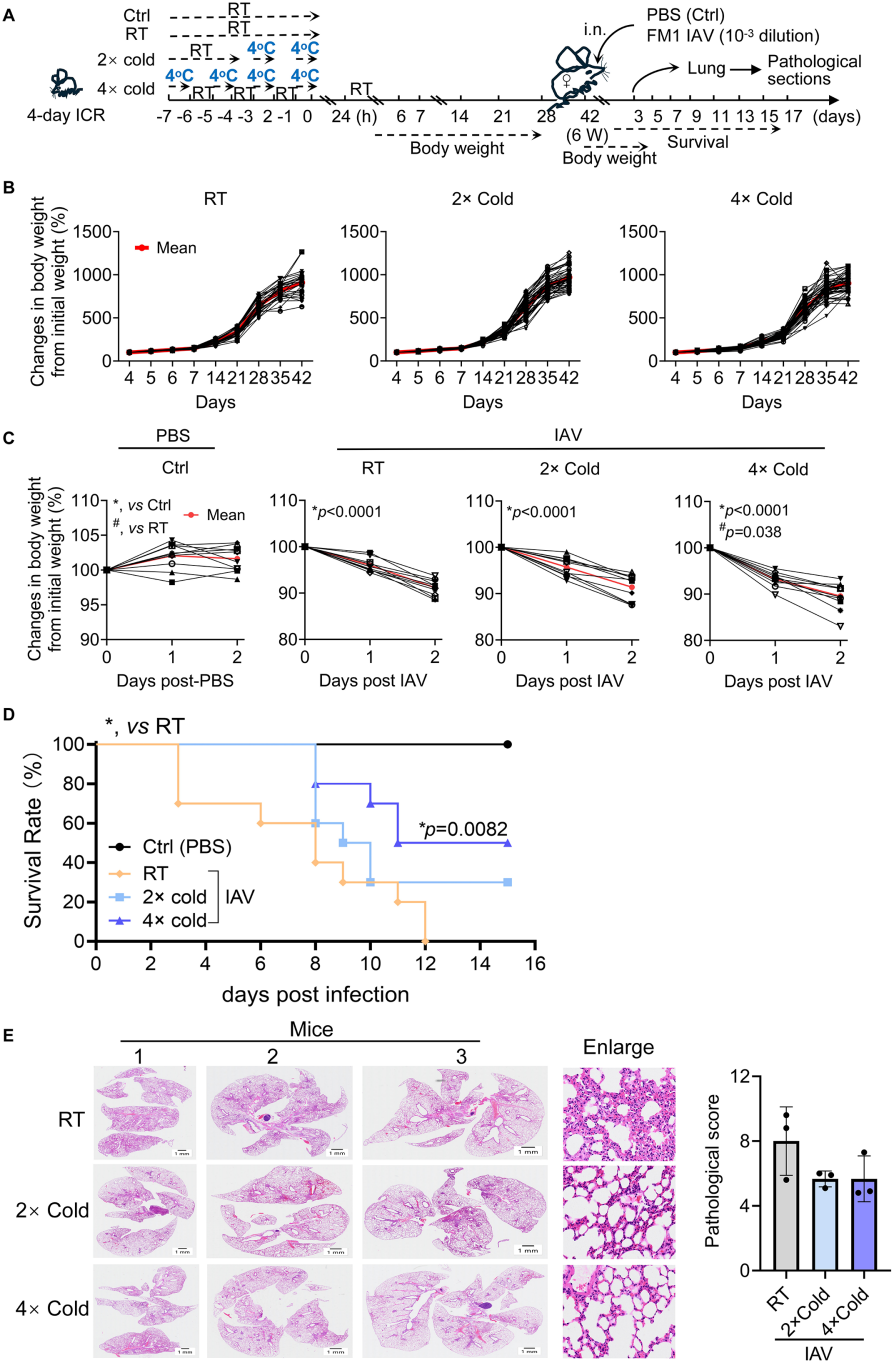
Frequency-selective effects of neonatal cyclic cold stress on anti-viral immunity against IAV infection in adulthood. **(A)** Experimental design. Four-day-old neonatal mice (mixed sex) were subjected to 2× or 4× cycles of cold stress (1 h at 4 °C followed by 1 h at RT per cycle), then raised normally until 6 weeks of age. At adulthood, only female mice (n=10 in each group) were selected for intranasal infection with FM1 influenza A virus (FM1 IAV). Body weight and survival were monitored post-infection. **(B)** Body weight before IAV infection. **(C)** Body weight changes after IAV infection. **(D)** Survival curves post-IAV infection. At least two independent experiments. **(E)** Lung histopathology on day 3 post IAV infection in adult mice following neonatal cyclic cold stress. Representative H&E- stained lung sections are shown on the left, and quantitative pathological scores are shown on the right. Each dot represents one mouse, with three mice per group.

We next asked whether neonatal cold stress could influence anti-viral immunity when the cold-stress neonatal mice reached adulthood. Four-day-old neonatal mice were subjected to 2× or 4× stress, then raised normally until 6 weeks of age, when they were intranasally infected with IAV (**Figure 4A**). No body weight differences were observed among groups prior to infection (**Figure 4B**). Following IAV infection, mice in all groups lost body weight, most markedly in the 4× stress group (**Figure 4C**). Survival analysis revealed that control mice began dying on day 3 post-infection and all died by day 12. In contrast, mice in both 2× and 4× stress began dying on day 8. By day 15, the survival rate was 30% in the 2× stress and 50% in the 4× stress (**Figure 4D**). To further evaluate the long-term protective effect, lung histopathological changes were assessed on day 3 post IAV infection. As shown in **Figure 4E**, both the 2× and 4× stress showed a trend toward reduced lung pathological changes compared to Ctrl, but no significant differences were found, possibly due to the small sample size (n=3 per group). Together, these findings reveal that neonatal cold stress can reprogram anti-viral immunity in a depending on frequency of cold stress and the age of the host: 2× stress protects acutely, whereas 4× stress, despite lacking acute benefit, confers superior long-term survival in adulthood. The underlying mechanisms deserve further investigation.

### Cyclic cold stress induces frequency-selective and dynamic alterations in the expression of metabolic-immune signaling molecules in mice

**Figure 5:**
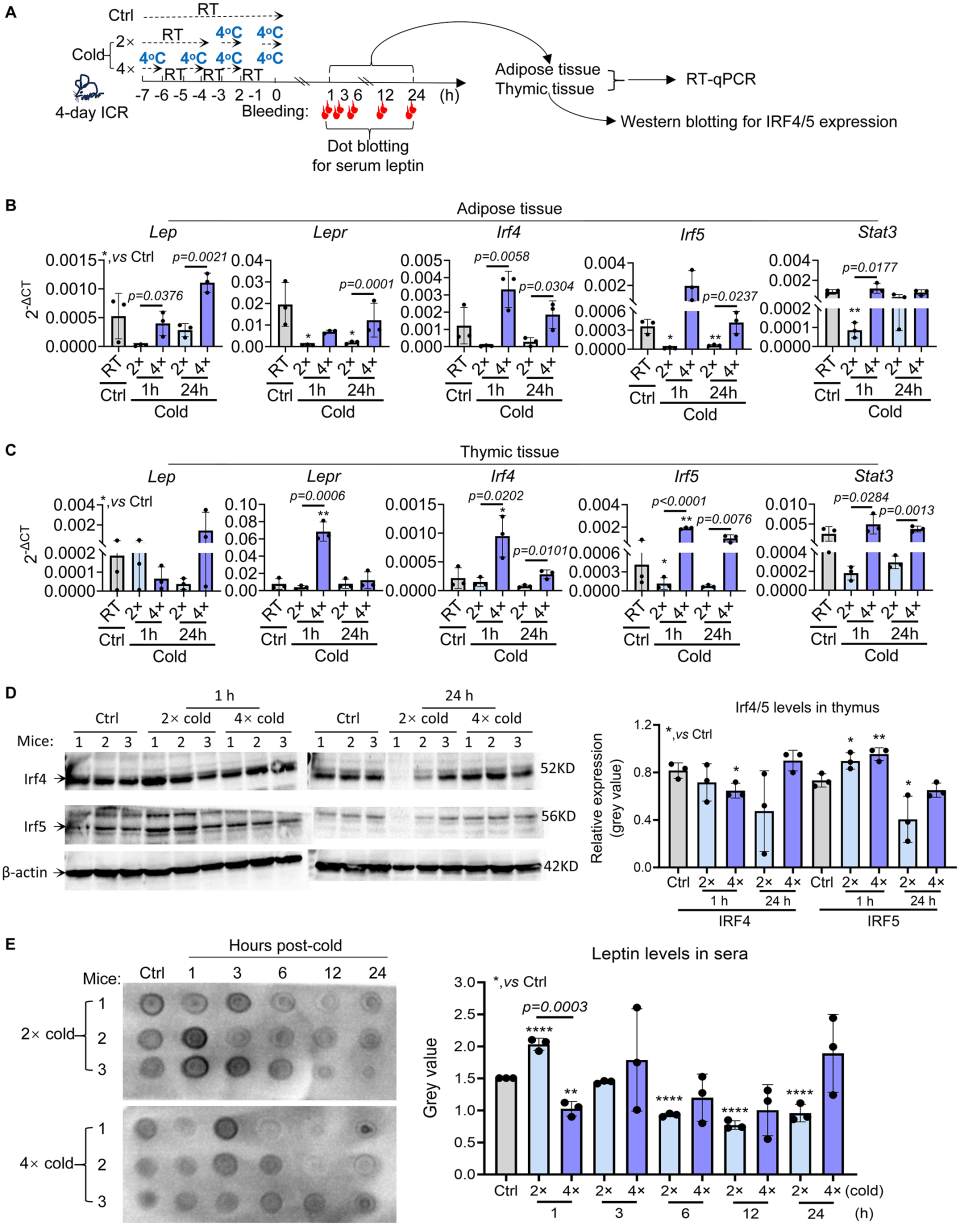
Cold stress differentially modulates systemic leptin signaling and the metabolic-immune axis based on the dose and timing of exposure. **(A)** Experimental procedure. **(B)** mRNA levels of *Lep*, *Lepr*, *Irf4*, *Irf5*, and *Stat3* signaling molecules in adipose tissue at 1 h and 24 h post-cold stress. **(C)** mRNA levels of *Lep*, *Lepr*, *Irf4*, *Irf5*, and *Stat3* signaling molecules in thymic tissue at 1 h and 24 h post-cold stress. **(D)** Western blot analysis of Irf4 and Irf5 protein levels in thymic tissue at 1 h and 24 h post-cold stress. **(E)** Dot blot analysis of serum Leptin protein levels at indicated time points post-cold stress. Each data point represents one mouse. ^*^p < 0.05; ^**^p < 0.01; ^***^p < 0.001; ^****^p < 0.0001.

To investigate the potential mechanisms by which different cyclic cold stress affects thymic T cells and anti-infection immunity in neonatal mice, we focused on the expression changes of *Lep, Lepr, Irf4/5*, *Stat3*, and inflammatory factors, based on prior clues from our zebrafish study. Using the 2× and 4× stress paradigms (**Figure 5A**), RT-qPCR analysis revealed a divergent pattern. In both adipose and thymic tissues, 2× stress generally suppressed the mRNA levels of *Lep, Lepr, Irf4, Irf5*, and *Stat3* at 1 h and 24 h post-stress. In contrast, 4× stress significantly enhanced the expression of these genes. Notably, in the thymus, the expression levels of *Lepr*, *Irf4* and *Irf5* were already significantly higher than those in the Ctrl as early as 1 h after the initiation of 4× stress (**Figures 5B, C**). Regarding inflammatory factors, compared to the Ctrl, the 2× stress showed a general downregulation of *Nf-kb, Tnf-α/β,* and *Ifn-α/β* mRNA levels in both adipose and thymic tissues, with the exception of elevated *Il-6* and *Ifn-r* mRNA specifically in the thymus. Conversely, the 4× stress exhibited a broad upregulation of these genes compared to 2× stress. However, histopathological examination of the thymus revealed no significant differences in either stress group compared to the Ctrl (**Supplementary Figure 3**). We next assessed changes in Irf4 and Irf5 protein levels in mouse thymic tissue by Western blotting. The results showed that, compared to Ctrl, the 2× stress exhibited no significant change in Irf4 levels, whereas Irf5 levels showed an increasing trend at 1 h and a significant decrease by 24 h post-stress. In the 4× stress, Irf4 levels were significantly reduced at 1 h, with a trend toward increase at 24 h, whereas Irf5 levels were significantly elevated at 1 h but trended downward by 24 h (**Figure 5D**). Serum leptin protein levels exhibited dynamic alterations. In the 2× stress, leptin levels showed a transient but significant increase at 1 h post-stress, followed by a sustained low concentration thereafter. In contrast, the 4× stress displayed a dysregulated oscillatory pattern, characterized by an initial decrease followed by a subsequent increase (**Figure 5E**). Notably, this variability in leptin levels occurred in the absence of significant body weight differences among the groups at the corresponding time point shown in **Figure 3B**, and potential sex-specific effects were not assessed as mixed-sex litters were used. Together, these mouse data indicate that cold stress at a certain frequency can precondition metabolic-immune responses. To confirm this, we turned to gain- and loss-of-function zebrafish models to establish causality.

### Genetic overexpression of *Irf4 or Irf5* establishes distinct metabolic-immune baselines and alters the response to cold stress in zebrafish

**Figure 6:**
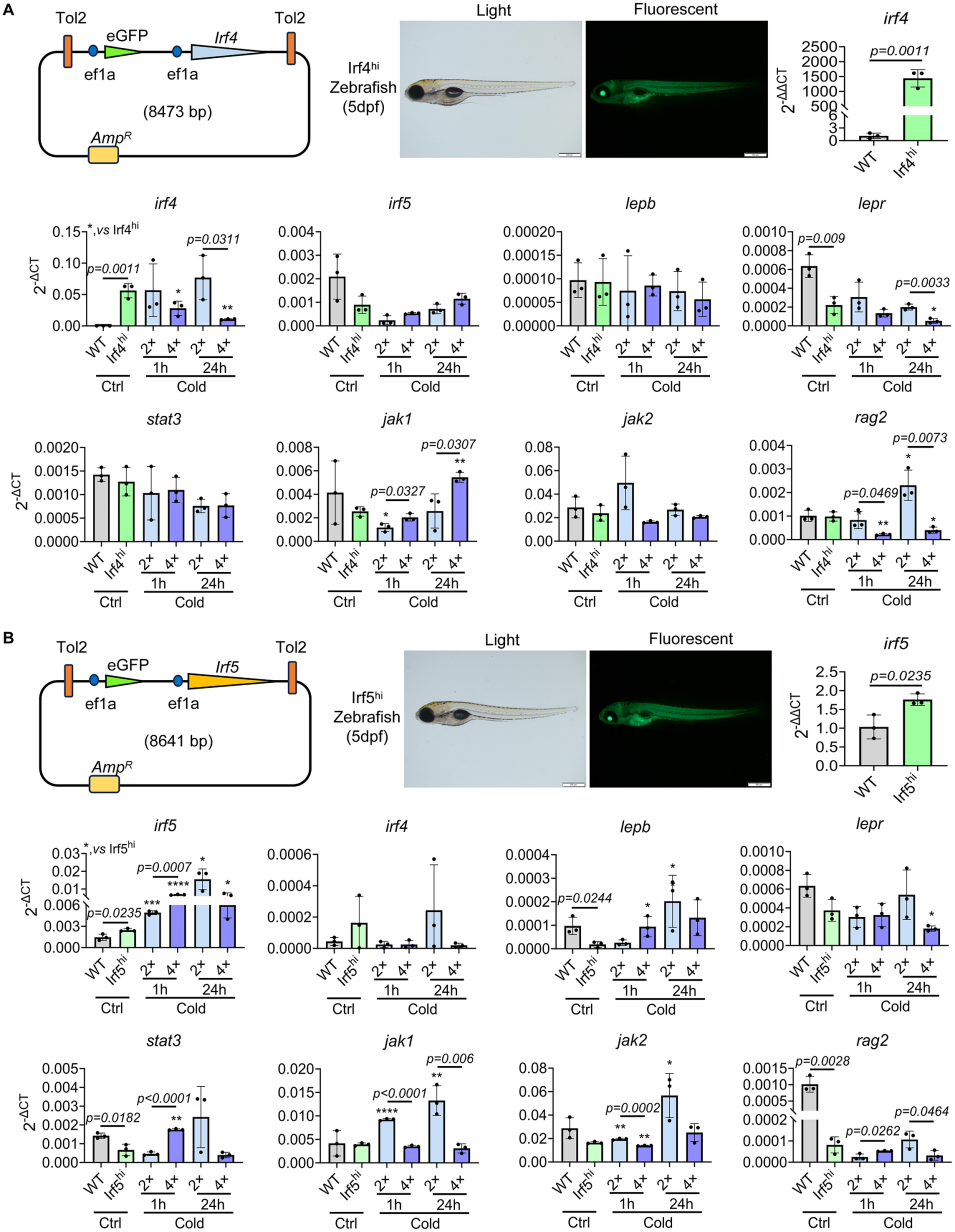
Effects of Irf4/5 transgenesis on the expression of metabolic-immune genes in zebrafish larvae under basal conditions and following cold stress. *Irf4* and *irf5* transgenic zebrafish were generated using their respective transgenic plasmid constructs and verified by fluorescence microscopy and RT-qPCR. At 4 dpf, transgenic larvae were exposed to 2× or 4× cyclic cold stress. 24 h later, mRNA levels of *irf4/5*, *lepb*, *lepr*, *stat3*, *jak1/2*, and *rag2* were assessed by RT-qPCR using whole-larva samples, each pooled from 10 larvae. **(A)** Plasmid map of *irf4* transgene, fluorescence images of *irf4* transgenic zebrafish, and changes in mRNA levels of *irf4* as well as metabolism-immunity-related genes in *irf4* transgenic zebrafish. **(B)** Plasmid map of *irf5* transgene, fluorescence images of *irf5* transgenic zebrafish, and changes in mRNA levels of *irf5* as well as metabolism-immunity-related genes in *irf5* transgenic zebrafish. ^*^p < 0.05; ^**^p < 0.01; ^***^p < 0.001; ^****^p < 0.0001.

To delineate the *in vivo* functions of IRF4 and IRF5 in the cold stress response, we generated *irf4* and *irf5* overexpressing transgenic zebrafish lines, using independent ef1a promoters to drive each gene of interest along with a co-integrated eGFP reporter. Stable transgenic larvae were selected based on the exhibition of strong and ubiquitous GFP fluorescence, which indicates germline transmission. RT-qPCR verified significant overexpression, with *irf4* mRNA increased approximately 1500-fold and *irf5* mRNA increased 2-fold compared to wild-type (**Figures 6A, B**, top row). Using these transgenic lines, we found that *irf4* or *irf5* transgenesis alone conferred distinct metabolic-immune baselines. Specifically, *irf4* overexpression led to an obvious reduction in basal *lepr* levels, whereas *irf5* overexpression resulted in significant downregulation of basal *lepb, stat3,* and *rag2* levels (**Figure 6**). Upon application of cyclic cold stress, the transgenic larvae with two genetic backgrounds exhibited markedly divergent responses. In *irf4* overexpressing larvae, cold stress (particularly the 4× stress) triggered a broadly suppressive effect, leading to reduced expression of *irf4* itself, *lepr,* and *rag2*. Meanwhile, *jak1* expression initially decreased and then increased, whereas the expression levels of *lepb, stat3, jak2* and endogenous *irf5* showed no significant difference compared to Ctrl (**Figure 6A**). In the *irf5* overexpressing larvae, the 2× stress reversed its baseline suppressed state, leading to recovery of *lepb*, *lepr, stat3,* and *rag2* expression at 24 h, accompanied by significantly elevated *jak1* and *jak2* at both 1 h and 24 h. Under 4× stress, *lepb* and *stat3* were significantly increased at 1 h, whereas *lepr* was significantly reduced at 24 h. Notably, *irf5* itself was significantly upregulated under both 2× and 4× stress conditions, while endogenous *irf4* expression showed no significant change in the *irf5* transgenic larvae (**Figure 6B**). Together, these findings indicate that although both IRF4 and IRF5 can reset distinct metabolic-immune baselines, only IRF5 modulates the transcriptional response to cold stress in a frequency-selective manner, as reflected by its dynamic regulation of *lepb, stat3,* and *rag2*.

### Irf5 deficiency abrogates frequency-selective thymic reprogramming induced by cyclic cold stress in zebrafish

**Figure 7:**
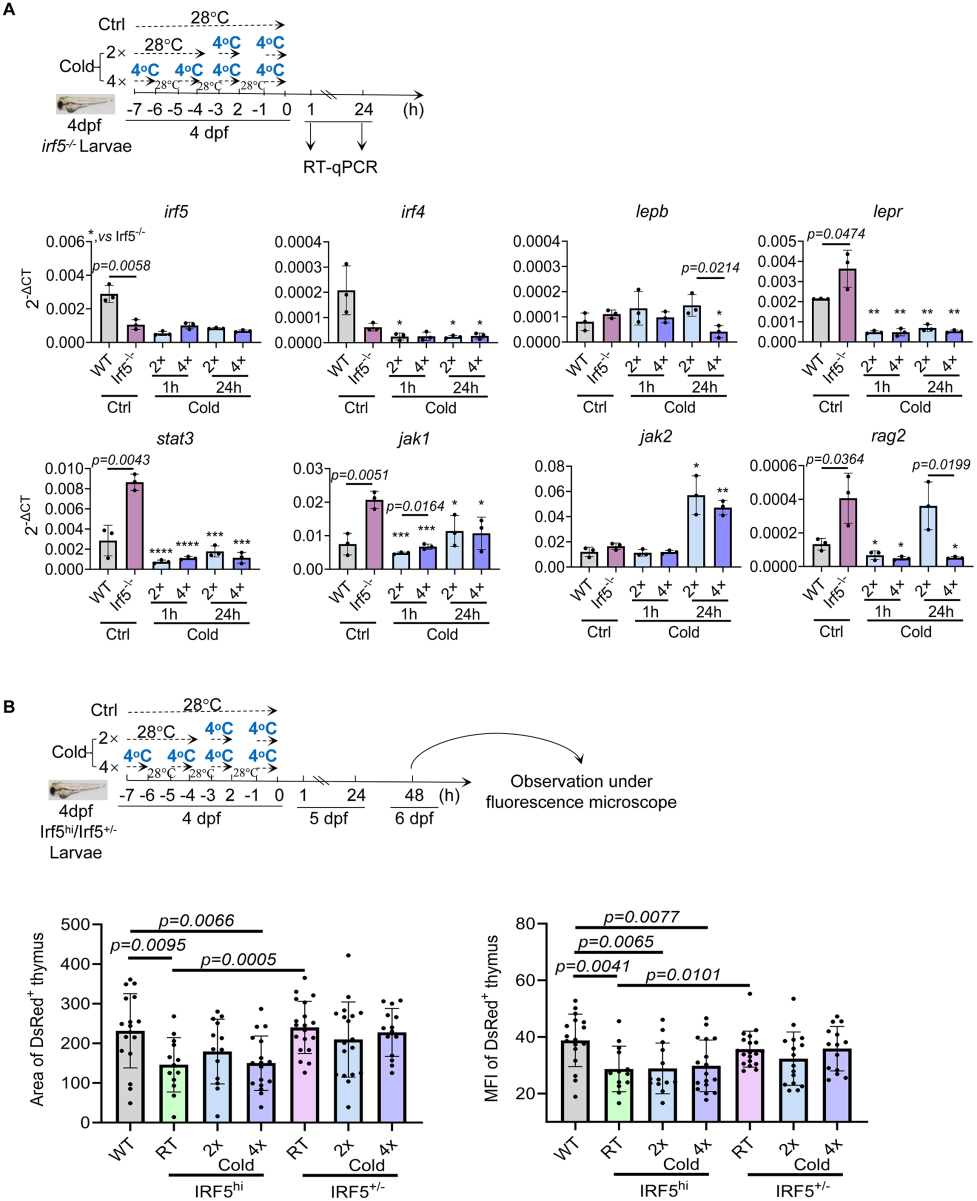
Irf5 deficiency alters the metabolic-immune baseline and post-cold stress thymic development in zebrafish larval. **(A)** mRNA levels of *irf4/5*, *lepb*, *lepr*, *stat3*, *jak1/2*, and *rag2* in whole fish of *irf5* knockout zebrafish before and after cold stress. Wild-type and *irf5*^-/-^ larvae reared at 28 °C served as controls. Each point in the mRNA expression graphs represents a pooled sample of 10 zebrafish. **(B)** Thymic development in *irf5*^hi^ and *irf5*^-/-^ zebrafish larvae following the same cold stress regimens. Thymic development was assessed based on the fluorescence area and mean fluorescence intensity (MFI) of the DsRed^+^ thymus. Wild-type (WT) zebrafish reared at 28 °C served as controls. Each point in the thymic fluorescence area and MFI graphs corresponds to one zebrafish larva. ^*^p < 0.05; ^**^p < 0.01; ^***^p < 0.001; ^****^p < 0.0001.

Given the above findings suggesting that IRF5 serves as a key metabolic-immune setter that programs the organism’s response to cold stress in a frequency-selective manner, we next utilized an *irf5* knockout (*irf5^-/-^*) zebrafish model. This allowed us to directly test IRF5 is necessity for the different effects of 2× versus 4× cold stress. We first examined, by RT-qPCR, the mRNA levels of leptin signaling molecules*, irf4/5* and *rag2* in whole-body tissues of *irf5^-/-^* zebrafish at 24 h after 2× or 4× cold stress, using wild-type (WT) and *irf5^-/-^* larvae reared at 28 °C as baseline controls. The results showed that, compared to WT fish, *irf5* deficiency significantly elevated the mRNA levels of *lepr, stat3, jak1,* and *rag2*, while *lepb* and *jak2* mRNA levels also showed increasing trends. Only *irf4* mRNA levels, similar to *irf5*, remained significantly lower than those in WT fish. Interestingly, after cold stress, with the exception of increased *jak2* mRNA levels at 24 h under both 2× and 4× stress and increased *rag2* mRNA levels under 2× stress, the changes in the other mRNA levels did not exceed those observed in the *irf5^-/-^* larval (**Figure 7A**). We then compared thymic development in *irf5^-/-^* zebrafish at 48 h (6 dpf) with that in *irf5* overexpressing (*irf5^hi^*) zebrafish. Compared to WT fish, *irf5* overexpression suppressed thymic development, as evidenced by markedly reduced fluorescence area and MFI, whereas *irf5* knockout reversed this phenotype, resulting in thymic development similar to that of WT zebrafish. At 6 dpf after 2× or 4× cold stress, *irf5^hi^* fish still exhibited very low thymic fluorescence area and MFI. The 4× stress showed even lower levels, similar to those in *irf5^hi^* larvae without cold stress. In contrast, *irf5^-/-^* zebrafish showed no obvious changes after either 2× or 4× stress. All groups remained similar to *irf5^-/-^* larvae without cold stress (**Figure 7B**). These gain- and loss-of-function experiments reveal that IRF5 is causally required for frequency-selective thymic reprogramming induced by cyclic cold stress. IRF5 deficiency abrogated thymic development-related responses to 2× and 4× stress, whereas IRF5 overexpression altered baseline thymic development and frequency-selective transcriptional responses. Together, these data demonstrate that IRF5 acts as a critical causal mediator downstream of cold stress frequency sensing.

## Discussion

Environmental stress during early life is recognized for shaping both immediate and long-term immune characteristics (67). However, the impact of cold stress, one of the most common physical environmental stressors, on early-life thymic development and T cell differentiation remains poorly understood. In this study, using zebrafish and mouse models, we found that cold stress frequency is a critical parameter, with 2× and 4× cyclic cold stresses exerting opposing effects on thymic development and T cell composition. Interestingly, the cold stress preconditioning translated into resilience in mice that matured from neonates having experienced cyclic cold stresses, protecting them against lethal influenza infection. Specifically, 2× stress confers acute protection, whereas 4× stress unexpectedly enhanced long-term survival following adult infection. Mechanistically, the resilience was traced to the leptin signaling pathway and its associated transcription factors IRF4 and IRF5 in cross-species analyses. Gain- and loss-of-function studies in zebrafish reveal that it is IRF5, not IRF4, that mediates the resilience. The complementary use of zebrafish and mouse models at an immature thermoregulatory stage (4 days) strengthens the cross-species generalizability of our findings. The choice of 4 °C as the cold stress temperature was based on developmental matching between species. It is known that yolk sac dependent larval stages exhibit enhanced resilience to ambient temperature stress (68, 69), and 4 dpf zebrafish (yolk sac dependent) tolerate 4 °C for up to 6 h without mortality (**Supplementary Figure 1**). In contrast, 4-day-old neonatal mice are poikilothermic at this stage and, without maternal care, cannot maintain core body temperature, which rapidly declines to ambient levels (70, 71).Thus, both species at 4 days of age depend on maternal nutrition and have limited thermoregulatory capacity, making them developmentally comparable. Although adult mice and zebrafish exhibit different cold stress mechanisms, the functional poikilothermic state of 4-day-old mice (63, 64) bridges this gap, enabling us to probe evolutionarily conserved pathways governing thymic programming.

Our investigation started with the observation that a single prolonged cold stress reversibly delays thymic development in zebrafish larvae and alters expression of genes enriched in the adipocytokine signaling pathway, with leptin and Irf4/5 as potential nodes. This led us to test whether a cyclic short-duration cold stress could induce frequency-related effects. Indeed, in zebrafish larvae, 2× stress increased the proportion of DsRed^+^ thymocytes, whereas 4× stress decreased it, accompanied by opposing expression patterns of *lepb, lepr, irf4/5*, and *stat3*. Extending these findings to a mammalian model, we examined neonatal mice and found that the impact of cyclic cold stress on thymic T cell composition is also varies with the number of cycles. Notably, 2× stress promoted thymocyte maturation, as reflected by increased CD3 expression on DP and CD4 SP cells, whereas 3× and 4× stress suppressed CD3 expression on DP, CD4 SP, and CD8 SP cells, with 4× stress showing more pronounced effects. This pattern reveals a frequency-selective window in which 2× stress drives opposing developmental outcomes compared to 3× and 4× stress, despite 1× stress having little effect. This window effect suggests that the immunomodulatory role of cold stress on thymic T cells operates within specific effective frequency ranges. One explanation is that different frequencies activate neuroendocrine signals of varying intensity or duration; only when the signal falls within a certain effective window can it be translated into measurable phenotypic changes, and exceeding this threshold may reverse the direction of immune programming. Notably, our analysis did not further subdivide DN cells into the early progenitor stages DN1–DN4 (72). While further sub-division using CD25 and CD44 would provide additional resolution of early thymocyte development, this was not feasible in the current study due to the low proportion of DN cells (<5% of total thymocytes) and limited cell numbers from individual neonatal thymus. Further studies incorporating DN staging will be valuable for a more detailed understanding of early thymocyte development. Additionally, our single time-point assessment (24 h) may have missed earlier or later dynamic changes.

The mechanism by which 4× stress reduces the proportion of thymic DP cells appears to involve selective regulation of cell fate rather than direct cytotoxicity, as evidenced by the absence of apoptosis or necrosis in the examined thymic tissue. The selective regulation induced by 4× stress may lead to a significantly increased proportion of CD4 SP cells, impede CD8 SP differentiation or survival, or alter the balance of DP lineage commitment toward CD4 SP versus CD8 SP. Thus, the effects of 4× stress likely involve fine-tuned regulation of cell fate decisions rather than simple numerical reduction. Importantly, this fine-tuned regulation may contribute to the strengthened antiviral function in mice that matured from neonates having experienced either 2× or 4× cold stress. It has been reported that BAL from 2-day-old neonatal mice contains few, if any, T cells (66). In our study, CD3^+^ T cells comprised less than 3% of total BAL cells at day 7 post-infection. This low proportion suggests that a small number of T cells do begin to appear in the alveolar space by 12 days of age in mice. Within this limited population, the proportion of DP cells was higher in the 4× stress group than in the 2× stress group. These findings raise the possibility that different cold stress frequencies may influence the trafficking or retention of immature T cells in the pulmonary compartment. Given the overall low T cell abundance, however, these observations should be interpreted with caution. Future studies examining T cell composition in lung tissue homogenates would help more accurately assess local immune responses and their contribution to the differential antiviral outcomes.

The most intriguing finding is that neonatal cold stress preconditioning confers frequency-selective resistance against lethal influenza. Neonatal 2× stress protects against acute IAV infection, but this benefit does not translate into a statistically significant survival advantage upon adult challenge beyond a trending prolongation relative to Ctrl. In contrast, neonatal 4× stress offers no acute protection but unexpectedly confers a statistically significant long-term survival advantage when mice are challenged as adults. Both stress groups also exhibit reduced lung pathology following adult infection compared to Ctrl. Mechanistically, we propose that 4× stress may trigger trained immunity in neonatal mice, involving epigenetic or metabolic adaptation. Previous studies have demonstrated that prior exposure to infections, vaccinations, or environmental stressors can durably modulate immune responses through trained immunity (73). Supporting this notion, neonatal mice subjected to 4× stress exhibit a significant increase in the proportion and number of thymic CD4 SP cells as shown in **Figure 2**. As we know, CD4 SP cells represent a heterogeneous population that encompasses various functionally distinct subsets, including naïve CD4^+^ T cells and regulatory T cells (Tregs). Neonatal CD4^+^ T cells can form long-lived memory cells (74), and clones generated early in life persist and dominate the memory pool (75). Moreover, early-life exposure to hormetic stressors has been reported to shape the CD4^+^ T cell transcriptome, influencing subsequent proliferation, differentiation, and mitochondrial dynamics later in life (76). Thus, the expansion of thymic CD4 SP cells following neonatal 4× stress may provide a cellular substrate for long-term antiviral protection in adulthood, potentially by enlarging the CD4^+^ T cell pool or by establishing an enhanced regulatory milieu that limits immunopathology during infection. A striking observation is that mice subjected to neonatal 4× stress exhibit the most pronounced body weight loss following adult infection yet achieve the best survival rate. This paradox may reflect a trade-off between antiviral defense and metabolic demands (73, 76). Future studies examining epigenetic modifications, metabolic reprogramming of immune cells, and the fate of neonatal-derived CD4 SP cells into adulthood are required to dissect the underlying mechanisms.

In this study, we found that moderate (2×) cold stress triggers a sharp but transient rise in leptin signaling followed by rapid return to baseline, accompanied by an initial broad suppression of inflammatory cytokines. In contrast, excessive (4×) cold stress induces dysregulated oscillations in leptin signaling and its downstream JAK/STAT3 pathway, and IRF5 expression. A notable complexity arising from the transgenic and knockout zebrafish models is that mRNA levels of core signaling molecules such as *jak2* and *stat3* do not always correlate directly with those of *lepb* or *lepr*. Thus, the functional outcome of cold stress is likely determined by integrated signaling flux through the entire network rather than by any single transcript. While previous understanding emphasized the sympathetic nervous system β-adrenergic receptor axis or the hypothalamic-pituitary-adrenal axis as primary mechanisms regulating cold exposure responses (77–79), we propose that cold stress of differing intensities may activate distinct neuroendocrine-immune signaling pathways. Notably, the variability in leptin levels observed in this study occurred in the absence of significant body weight differences among the groups at the corresponding time point (**Figure 3B**). However, leptin levels are also known to be influenced by sex and circadian rhythm. In this study, mixed-sex neonatal litters were used, and potential sex-specific effects on leptin dynamics were not assessed. It should be noted that 4-day-old neonatal mice were exclusively breastfed; therefore, feeding status does not represent a confounding variable in this model, in contrast to adult mice. Additionally, all cold stress procedures and sampling were performed at consistent times of day across groups to minimize circadian influences. Future studies controlling for sex will be needed to further validate the leptin dynamics reported here.

Whole-fish RNA sequencing in zebrafish directed our mechanistic focus toward leptin signaling, with IRF4 and IRF5 as potential downstream nodes. We chose to study both in parallel based on independent literature: IRF4 is a critical transcription factor for T cell differentiation and thymic development (80); IRF5 links metabolism and immunity, particularly in promoting inflammatory responses and macrophage polarization. We observed similar induction patterns of both IRF4 and IRF5 in response to cold stress in zebrafish and mice. However, transgenic zebrafish overexpressing either *irf4* or *irf5* revealed a functional divergence. *Irf4* overexpression established a distinct metabolic-immune baseline (reduced basal *lepr*) but, upon cold stress, exhibited a broadly suppressive response regardless of stress frequency. In contrast, *irf5* overexpression not only established a different baseline (reduced basal *lepb, stat3,* and *rag2*) but also displayed a striking frequency-selective pattern: 2× stress reversed the baseline-suppressed state, whereas 4× stress exerted additional inhibitory effects on lepr expression. This functional specialization of IRF5, not observed for IRF4, led us to focus on IRF5 for loss-of-function studies. Using *irf5* knockout zebrafish, we directly tested the necessity of IRF5. *Irf5* deficiency significantly elevated baseline mRNA levels of *lepr, stat3, jak1,* and *rag2*, and, most importantly, largely abrogated stress-induced molecular and thymic developmental alterations. Unlike wild-type fish, *irf5^-/-^* zebrafish showed no obvious changes in thymic development after either 2× or 4× cold stress. These loss-of-function data, together with the gain-of-function data from *irf5* transgenic zebrafish, collectively demonstrate that IRF5 is both sufficient and necessary for mediating frequency-selective thymic reprogramming induced by cyclic cold stress in zebrafish. In *irf5* transgenic and knockout zebrafish, baseline phenotypes revealed additional insights beyond cold stress responses. Specifically, *irf5* overexpression suppressed baseline expression of *lepb, stat3,* and *rag2*, whereas *irf5* deficiency elevated baseline expression of *lepr, stat3, jak1,* and *rag2*. These findings suggest that IRF5 is associated with an inhibitory influence on the leptin-STAT3 axis and thymic progenitor proliferation under steady-state conditions, functioning as a molecular brake that constrains baseline thymic developmental capacity. This interpretation is consistent with the observation that *irf5*-overexpressing fish showed a suppressed response to both 2× and 4× cold stress, whereas *irf5* knockout fish exhibited little additional change upon stress, as if the pathway was already derepressed at baseline. Thus, we propose that IRF5 calibrates both the baseline setpoint and the frequency-selective stress responsiveness of the thymus. This dual role does not conflict with its stress-induced function; rather, it positions IRF5 as a key integrator of steady-state homeostasis and environmental challenge. In mice, we observed that frequency-selective thymic effects are correlated with dynamic changes in leptin signaling components (serum leptin by dot blot) and IRF4/5 protein levels (by Western blot), without direct perturbation of IRF5 or measurement of phospho-STAT3. While these correlative findings are consistent with the possibility that similar mechanisms operate in mammals, they do not prove causality. Therefore, we propose a working model based on the zebrafish data. In this model, IRF5 serves as a critical molecular setpoint that translates cold stress frequency into immune instructions via the leptin STAT3 axis. The relevance of this axis in mammals remains to be experimentally tested in future studies using genetic or pharmacological perturbations in mice.

This study has several limitations. First, the upstream molecular events from physical perception of cold stress remain to be explored. Second, our anti-viral immunity analysis relied exclusively on mouse models, precluding direct functional comparison with zebrafish. Although the mouse is the established model for influenza A virus infection, the lack of a parallel zebrafish infection model limits cross-species functional validation. Future work establishing zebrafish infection models using fish-specific viruses, such as SVCV or VHSV, would allow direct assessment of whether the observed frequency-selective thymic programming translates into protective immunity across species. Third, the specific cellular executors responsible for the protective effect of 2× stress have not been identified, nor has the impact of this early-life programming on responses to other viral infections in adulthood. Fourth, the causal requirement for IRF5 was established only in zebrafish. Thus, the leptin-IRF5 mechanism in mammals remains to be determined, requiring future experimental testing. Fifth, our 2× and 4× paradigms differ in both cycle number and total cold exposure duration (2 h vs. 4 h). Although the termination time of the last cold exposure was synchronized across groups to ensure comparable recovery durations, the observed effects may still reflect cumulative cold dose rather than cycle number alone. Future studies equalizing total cold exposure duration are needed to dissociate these variables.

## Data Availability

The raw data supporting the conclusions of this article will be made available by the authors, without undue reservation.

## References

[1] Henrick BM Rodriguez L Lakshmikanth T Pou C Henckel E Arzoomand A . Bifidobacteria-mediated immune system imprinting early in life. Cell. (2021) 184:3884–3898.e11. doi: 10.1016/j.cell.2021.05.030 34143954

[2] Serrano Matos YA Cano J Shafiq H Williams C Sunny J Cowardin CA . Colonization during a key developmental window reveals microbiota-dependent shifts in growth and immunity during undernutrition. Microbiome. (2024) 12:71. doi: 10.1186/s40168-024-01783-3 38589975 PMC11003143

[3] Renz H Brandtzaeg P Hornef M . The impact of perinatal immune development on mucosal homeostasis and chronic inflammation. Nat Rev Immunol. (2012) 12:9–23. doi: 10.1038/nri3112 22158411

[4] Tabilas C Smith NL Rudd BD . Shaping immunity for life: Layered development of CD8^+^ T cells. Immunol Rev Immunol Rev. (2023) 315:108–25. doi: 10.1111/imr.13185 36653953 PMC10205662

[5] Gluckman PD Hanson MA . Evolution, development and timing of puberty. Trends Endocrinol Metab Trends Endocrinol Metab. (2006) 17:7–12. doi: 10.1016/j.tem.2005.11.006 16311040

[6] Lapehn S Paquette AG . The placental epigenome as a molecular link between prenatal exposures and fetal health outcomes through the DOHaD hypothesis. Curr Envir Health Rpt. (2022) 9:490–501. doi: 10.1007/s40572-022-00354-8 35488174 PMC9363315

[7] Hales CN . Fetal and infant origins of adult disease. J Clin Pathol. (1997) 50:359. doi: 10.1136/jcp.50.5.359 9215113 PMC499932

[8] Barker DJP . The origins of the developmental origins theory. J Internal Med J Intern Med. (2007) 261:412–7. doi: 10.1111/j.1365-2796.2007.01809.x 17444880

[9] Hsu C-N Hou C-Y Hsu W-H Tain Y-L . Early-life origins of metabolic syndrome: Mechanisms and preventive aspects. IJMS Int J Mol Sci. (2021) 22:11872. doi: 10.3390/ijms222111872 34769303 PMC8584419

[10] Bach J-F . The hygiene hypothesis in autoimmunity: the role of pathogens and commensals. Nat Rev Immunol. (2018) 18:105–20. doi: 10.1038/nri.2017.111 29034905

[11] Rook GA . Regulation of the immune system by biodiversity from the natural environment: An ecosystem service essential to health. Proc Natl Acad Sci USA. (2013) 110:18360–7. doi: 10.1073/pnas.1313731110 24154724 PMC3831972

[12] Strachan DP . Hay fever, hygiene, and household size. BMJ. (1989) 299:1259–60. doi: 10.1136/bmj.299.6710.1259 2513902 PMC1838109

[13] Lim AI McFadden T Link VM Han S-J Karlsson R-M Stacy A . Prenatal maternal infection promotes tissue-specific immunity and inflammation in offspring. Science. (2021) 373:eabf3002. doi: 10.1126/science.abf3002 34446580

[14] Serhan N Abdullah NS Gheziel N Loste A Ekren R Labit E . Maternal stress triggers early-life eczema through fetal mast cell programming. Nature. (2025) 646:161–70. doi: 10.1038/s41586-025-09419-8 40866704 PMC12488486

[15] Cannon B Nedergaard J . Brown adipose tissue: Function and physiological significance. Physiol Rev Physiol Rev. (2004) 84:277–359. doi: 10.1152/physrev.00015.2003 14715917

[16] Bartelt A Bruns OT Reimer R Hohenberg H Ittrich H Peldschus K . Brown adipose tissue activity controls triglyceride clearance. Nat Med. (2011) 17:200–5. doi: 10.1038/nm.2297 21258337

[17] Van Marken Lichtenbelt WD Vanhommerig JW Smulders NM Drossaerts JMAFL Kemerink GJ Bouvy ND . Cold-activated brown adipose tissue in healthy men. N Engl J Med. (2009) 360:1500–8. doi: 10.1056/NEJMoa0808718 19357405

[18] Spiljar M Steinbach K Rigo D Suárez-Zamorano N Wagner I Hadadi N . Cold exposure protects from neuroinflammation through immunologic reprogramming. Cell Metab Cell Metab. (2021) 33:2231–2246.e8. doi: 10.1016/j.cmet.2021.10.002 34687652 PMC8570411

[19] Foxman EF Storer JA Fitzgerald ME Wasik BR Hou L Zhao H . Temperature-dependent innate defense against the common cold virus limits viral replication at warm temperature in mouse airway cells. Proc Natl Acad Sci USA. (2015) 112:827–32. doi: 10.1073/pnas.1411030112 25561542 PMC4311828

[20] Moriyama M Ichinohe T . High ambient temperature dampens adaptive immune responses to influenza A virus infection. Proc Natl Acad Sci USA. (2019) 116:3118–25. doi: 10.1073/pnas.1815029116 30718396 PMC6386664

[21] Klein L Kyewski B Allen PM Hogquist KA . Positive and negative selection of the T cell repertoire: what thymocytes see (and don’t see). Nat Rev Immunol. (2014) 14:377–91. doi: 10.1038/nri3667 24830344 PMC4757912

[22] Haynes BF Hale LP . The human thymus: A chimeric organ comprised of central and peripheral lymphoid components. Immunol Res. (1998) 18:175–92. doi: 10.1007/BF02788778 9951649

[23] Anderson MS Su MA . Aire and T cell development. Curr Opin Immunol Curr Opin Immunol. (2011) 23:198–206. doi: 10.1016/j.coi.2010.11.007 21163636 PMC3073725

[24] Hosokawa H Rothenberg EV . How transcription factors drive choice of the T cell fate. Nat Rev Immunol. (2021) 21:162–76. doi: 10.1038/s41577-020-00426-6 32918063 PMC7933071

[25] Hosoya T Kuroha T Moriguchi T Cummings D Maillard I Lim K-C . GATA-3 is required for early T lineage progenitor development. J Exp Med J Exp Med. (2009) 206:2987–3000. doi: 10.1084/jem.20090934 19934022 PMC2806453

[26] Gruver A Hudson L Sempowski G . Immunosenescence of ageing. J Pathol J Pathol. (2007) 211:144–56. doi: 10.1002/path.2104 17200946 PMC1931833

[27] Lynch HE Goldberg GL Chidgey A Van Den Brink MRM Boyd R Sempowski GD . Thymic involution and immune reconstitution. Trends Immunol Trends Immunol. (2009) 30:366–73. doi: 10.1016/j.it.2009.04.003 19540807 PMC2750859

[28] Ruiz Pérez M Vandenabeele P Tougaard P . The thymus road to a T cell: migration, selection, and atrophy. Front Immunol. (2024) 15:1443910. doi: 10.3389/fimmu.2024.1443910 39257583 PMC11384998

[29] Sempowski GD Hale LP Sundy JS Massey JM Koup RA Douek DC . Leukemia inhibitory factor, oncostatin M, IL-6, and stem cell factor mRNA expression in human thymus increases with age and is associated with thymic atrophy. J Immunol J Immunol. (2000) 164:2180–7. doi: 10.4049/jimmunol.164.4.2180 10657672

[30] Tournier J-N Mathieu J Mailfert Y Multon E Drouet C Jouan A . Chronic restraint stress induces severe disruption of the T‐cell specific response to tetanus toxin vaccine. Immunology. (2001) 102:87–93. doi: 10.1046/j.1365-2567.2001.01152.x 11168641 PMC1783154

[31] McDonald-McGinn DM Sullivan KE Marino B Philip N Swillen A Vorstman JAS . 22q11.2 deletion syndrome. Nat Rev Dis Primers. (2015) 1:15071. doi: 10.1038/nrdp.2015.71 27189754 PMC4900471

[32] Bautista JL Cramer NT Miller CN Chavez J Berrios DI Byrnes LE . Single-cell transcriptional profiling of human thymic stroma uncovers novel cellular heterogeneity in the thymic medulla. Nat Commun. (2021) 12:1096. doi: 10.1038/s41467-021-21346-6 33597545 PMC7889611

[33] Biggs SE Gilchrist B May KR . Chromosome 22q11.2 deletion (DiGeorge syndrome): Immunologic features, diagnosis, and management. Curr Allergy Asthma Rep. (2023) 23:213–22. doi: 10.1007/s11882-023-01071-4 36897497 PMC9999075

[34] Nikanorova AA Barashkov NA Nakhodkin SS Pshennikova VG Solovyev AV Romanov GP . The role of leptin levels in adaptation to cold climates. IJERPH Int J Environ Res Public Health. (2020) 17:1854. doi: 10.3390/ijerph17061854 32178438 PMC7143756

[35] Hick RW Gruver AL Ventevogel MS Haynes BF Sempowski GD . Leptin selectively augments thymopoiesis in leptin deficiency and lipopolysaccharide-induced thymic atrophy. J Immunol J Immunol. (2006) 177:169–76. doi: 10.4049/jimmunol.177.1.169 16785512 PMC1993881

[36] Zhang Y Proenca R Maffei M Barone M Leopold L Friedman JM . Positional cloning of the mouse obese gene and its human homologue. Nature. (1994) 372:425–32. doi: 10.1038/372425a0 7984236

[37] Lord GM Matarese G Howard JK Baker RJ Bloom SR Lechler RI . Leptin modulates the T-cell immune response and reverses starvation-induced immunosuppression. Nature. (1998) 394:897–901. doi: 10.1038/29795 9732873

[38] Girasol A Albuquerque GG Mansour E Araújo EP Degasperi G Denis RG . Fyn mediates leptin actions in the thymus of rodents. PloS One. (2009) 4:e7707. doi: 10.1371/journal.pone.0007707 19888448 PMC2766049

[39] Flier JS . Lowered leptin slims immune response. Nat Med. (1998) 4:1124–5. doi: 10.1038/2619 9771741

[40] Matarese G Moschos S Mantzoros CS . Leptin in immunology. J Immunol J Immunol. (2005) 174:3137–42. doi: 10.4049/jimmunol.174.6.3137 15749839

[41] Flores-Cordero JA Aranaz-Murillo A Vilariño-García T Pérez-Pérez A Izquierdo G Flores-Campos R . Leptin and leptin signaling in multiple sclerosis: A narrative review. NeuroMol Med. (2025) 27:19. doi: 10.1007/s12017-025-08842-4 40019662 PMC11870953

[42] De Rosa V Procaccini C Calì G Pirozzi G Fontana S Zappacosta S . A key role of leptin in the control of regulatory T cell proliferation. Immunity. (2007) 26:241–55. doi: 10.1016/j.immuni.2007.01.011 17307705

[43] Fantuzzi G Faggioni R . Leptin in the regulation of immunity, inflammation, and hematopoiesis. J Leukoc Biol. (2000) 68:437–46. doi: 10.1189/jlb.68.4.437 11037963

[44] Matarese G Leiter EH La Cava A . Leptin in autoimmunity: many questions, some answers. Tissue Antigens. (2007) 70:87–95. doi: 10.1111/j.1399-0039.2007.00886.x 17610413

[45] Lohoff M Mittrücker H-W Prechtl S Bischof S Sommer F Kock S . Dysregulated T helper cell differentiation in the absence of interferon regulatory factor 4. Proc Natl Acad Sci USA. (2002) 99:11808–12. doi: 10.1073/pnas.182425099 12189207 PMC129350

[46] Muroi S Naoe Y Miyamoto C Akiyama K Ikawa T Masuda K . Cascading suppression of transcriptional silencers by ThPOK seals helper T cell fate. Nat Immunol. (2008) 9:1113–21. doi: 10.1038/ni.1650 18776907

[47] Cao Y Li H Sun Y Chen X Liu H Gao X . Interferon regulatory factor 4 regulates thymocyte differentiation by repressing Runx3 expression. Eur J Immunol. (2010) 40:3198–209. doi: 10.1002/eji.201040570 21061442

[48] Setoguchi R Tachibana M Naoe Y Muroi S Akiyama K Tezuka C . Repression of the transcription factor Th-POK by Runx complexes in cytotoxic T cell development. Science. (2008) 319:822–5. doi: 10.1126/science.1151844 18258917

[49] Egawa T Littman DR . ThPOK acts late in specification of the helper T cell lineage and suppresses Runx-mediated commitment to the cytotoxic T cell lineage. Nat Immunol. (2008) 9:1131–9. doi: 10.1038/ni.1652 18776905 PMC2666788

[50] Zheng Y Zha Y Gajewski TF . Molecular regulation of T‐cell anergy. EMBO Rep EMBO Rep. (2008) 9:50–5. doi: 10.1038/sj.embor.7401138 18174897 PMC2246614

[51] Eguchi J Wang X Yu S Kershaw EE Chiu PC Dushay J . Transcriptional control of adipose lipid handling by IRF4. Cell Metab. (2011) 13:249–59. doi: 10.1016/j.cmet.2011.02.005 21356515 PMC3063358

[52] Al-Rashed F Sindhu S Arefanian H Al Madhoun A Kochumon S Thomas R . Repetitive intermittent hyperglycemia drives the M1 polarization and inflammatory responses in THP-1 macrophages through the mechanism involving the TLR4-IRF5 pathway. Cells. (2020) 9:1892. doi: 10.3390/cells9081892 32806763 PMC7463685

[53] Gallucci S Meka S Gamero AM . Abnormalities of the type I interferon signaling pathway in lupus autoimmunity. Cytokine. (2021) 146:155633. doi: 10.1016/j.cyto.2021.155633 34340046 PMC8475157

[54] Hedl M Yan J Abraham C . IRF5 and IRF5 disease-risk variants increase glycolysis and human M1 macrophage polarization by regulating proximal signaling and Akt2 activation. Cell Rep. (2016) 16:2442–55. doi: 10.1016/j.celrep.2016.07.060 27545875 PMC5165654

[55] Yang G Xi Z-X Wan Y Wang H Bi G . Changes in circulating and tissue angiotensin II during acute and chronic stress. Neurosignals. (1993) 2:166–72. doi: 10.1159/000109488 8004155

[56] Ishikawa C Senba M Barnes BJ Mori N . Constitutive expression of IRF-5 in HTLV-1-infected T cells. Int J Oncol. (2015) 47:361–9. doi: 10.3892/ijo.2015.3020 26004104

[57] Yan J Pandey SP Barnes BJ Turner JR Abraham C . T cell-intrinsic IRF5 regulates T cell signaling, migration, and differentiation and promotes intestinal inflammation. Cell Rep. (2020) 31:107820. doi: 10.1016/j.celrep.2020.107820 32610123 PMC7409536

[58] Suo C Dann E Goh I Jardine L Kleshchevnikov V Park J-E . Mapping the developing human immune system across organs. Science. (2022) 376:eabo0510. doi: 10.1126/science.abo0510 35549310 PMC7612819

[59] Wu J Ge D Zhong T Chen Z Zhou Y Hou L . IRF4 and STAT3 activities are associated with the imbalanced differentiation of T-cells in responses to inhalable particulate matters. Respir Res. (2020) 21:123. doi: 10.1186/s12931-020-01368-2 32448264 PMC7245756

[60] Shao Q Wang H Li S Zeng M Zhang S Yan X . IRF5 mediates artery inflammation in salt-sensitive hypertension by regulating STAT1 and STAT2 phosphorylation to increase ESM1 transcription: Insights from bioinformatics and mechanistic analysis. IJMS. (2025) 26:3722. doi: 10.3390/ijms26083722 40332339 PMC12027925

[61] García-Moreno D Tyrkalska SD Valera-Pérez A Gómez-Abenza E Pérez-Oliva AB Mulero V . The zebrafish: A research model to understand the evolution of vertebrate immunity. Fish Shellfish Immunol. (2019) 90:215–22. doi: 10.1016/j.fsi.2019.04.067 31039438

[62] Mestas J Hughes CCW . Of mice and not men: Differences between mouse and human immunology. J Immunol. (2004) 172:2731–8. doi: 10.4049/jimmunol.172.5.2731 14978070

[63] Tikhonova NS . Motor activity and heat loss in white mice in early ontogeny. Zh Evol Biokhim Fiziol. (1982) 18:532–5. 7148225

[64] Nagy ZM . Development of homeothermy in infant C3H mice. Bull Psychon Soc. (1993) 31:221–4. doi: 10.3758/BF03337329

[65] Gong J Liu J Ronan EA He F Cai W Fatima M . A cold-sensing receptor encoded by a glutamate receptor gene. Cell. (2019) 178:1375–1386.e11. doi: 10.1016/j.cell.2019.07.034 31474366 PMC6743979

[66] Lines JL Hoskins S Hollifield M Cauley LS Garvy BA . The migration of T cells in response to influenza virus is altered in neonatal mice. J Immunol. (2010) 185:2980–8. doi: 10.4049/jimmunol.0903075 20656925 PMC2924920

[67] Hong JY Medzhitov R . On developmental programming of the immune system. Trends Immunol. (2023) 44:877–89. doi: 10.1016/j.it.2023.09.004 37852863

[68] Hunt von Herbing I Pan FTC . Multiple stressors, allostasis and metabolic scaling in developing zebrafish. J Exp Biol. (2022) 225:jeb244095. doi: 10.1242/jeb.244095 36172880

[69] Long Y Song G Yan J He X Li Q Cui Z . Transcriptomic characterization of cold acclimation in larval zebrafish. BMC Genomics. (2013) 14:612. doi: 10.1186/1471-2164-14-612 24024969 PMC3847098

[70] Vinter J Hull D Elphick MC . Onset of thermogenesis in response to cold in newborn mice. Biol Neonate. (1982) 42:145–51. doi: 10.1159/000241588 7138988

[71] Wang H-Y Peng X-M Yang M Weng Y Yang X Zhan D . C5aR1-positive adipocytes mediate non-shivering thermogenesis in neonatal mice. Iscience. (2024) 27:111261. doi: 10.1016/j.isci.2024.111261 39758991 PMC11700647

[72] Pénit C Lucas B Vasseur F . Cell expansion and growth arrest phases during the transition from precursor (CD4^-^8^-^) to immature (CD4^+^8^+^) thymocytes in normal and genetically modified mice. J Immunol. (1995) 154:5103–13. doi: 10.4049/jimmunol.154.10.5103 7730616

[73] Tomalka JA Suthar MS Diamond MS Sekaly RP . Innate antiviral immunity: how prior exposures can guide future responses. Trends Immunol. (2022) 43:696–705. doi: 10.1016/j.it.2022.07.001 35907675

[74] Soares AP Kwong Chung CKC Choice T Hughes EJ Jacobs G van Rensburg EJ . Longitudinal changes in CD4(+) T-cell memory responses induced by BCG vaccination of newborns. J Infect Dis. (2013) 207:1084–94. doi: 10.1093/infdis/jis941 23293360 PMC3583271

[75] Bullock ME Hogan T Williams C Morris S Nowicka M Sharjeel M . The dynamics and longevity of circulating CD4+ memory T cells depend on cell age and not the chronological age of the host. PloS Biol. (2024) 22:e3002380. doi: 10.1371/journal.pbio.3002380 39137219 PMC11321570

[76] Burke CG Myers JR Boule LA Post CM Brookes PS Lawrence BP . Early life exposures shape the CD4+ T cell transcriptome, influencing proliferation, differentiation, and mitochondrial dynamics later in life. Sci Rep. (2019) 9:11489. doi: 10.1038/s41598-019-47866-2 31391494 PMC6686001

[77] Abe Y Rozqie R Matsumura Y Kawamura T Nakaki R Tsurutani Y . JMJD1A is a signal-sensing scaffold that regulates acute chromatin dynamics via SWI/SNF association for thermogenesis. Nat Commun. (2015) 6:7052. doi: 10.1038/ncomms8052 25948511 PMC4432656

[78] Kim H-G Lee J-S Han J-M Lee J-S Choi M-K Son S-W . Myelophil attenuates brain oxidative damage by modulating the hypothalamus-pituitary-adrenal (HPA) axis in a chronic cold-stress mouse model. J Ethnopharmacol. (2013) 148:505–14. doi: 10.1016/j.jep.2013.04.046 23665312

[79] Lowell BB Spiegelman BM . Towards a molecular understanding of adaptive thermogenesis. Nature. (2000) 404:652–60. doi: 10.1038/35007527 10766252

[80] Nam S Lim J-S . Essential role of interferon regulatory factor 4 (IRF4) in immune cell development. Arch Pharm Res. (2016) 39:1548–55. doi: 10.1007/s12272-016-0854-1 27826752

