# Supplementary material for: Frequency-selective thymic programming by early-life cold stress requires interferon regulatory factor 5
Source: Front Immunol. 2026 Jul 15;17:1851219. doi: 10.3389/fimmu.2026.1851219 (PMC13416564; doi:10.3389/fimmu.2026.1851219)
Supplement: Supplementary file 1 [file DataSheet1.pdf]

Supplements

Results

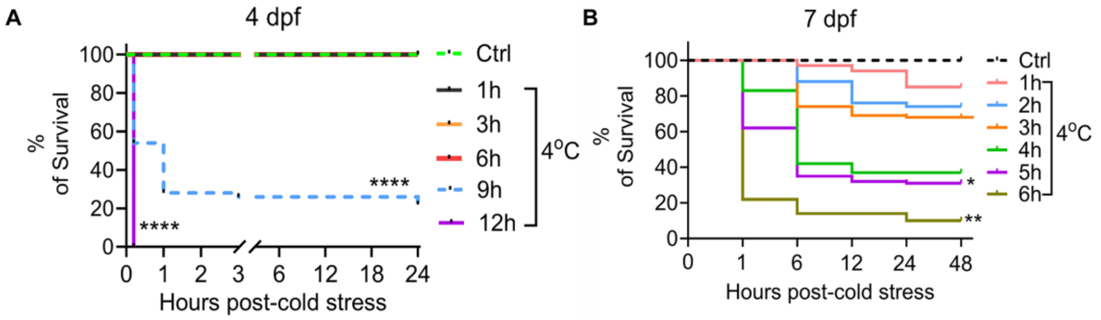

Figure S1. Stage-dependent cold tolerance of zebrafish larvae. (A) Survival of 4 dpf zebrafish larvae after continuous 4°C exposure for various duration. n=50 in each group. **(B)** Survival of 7 dpf zebrafish larvae after continuous 4°C exposure for various duration. n = 100 in each group. *, *vs* Ctrl, $^{*}p<0.05$, $^{**}p<0.01$, $^{****}p<0.0001$.

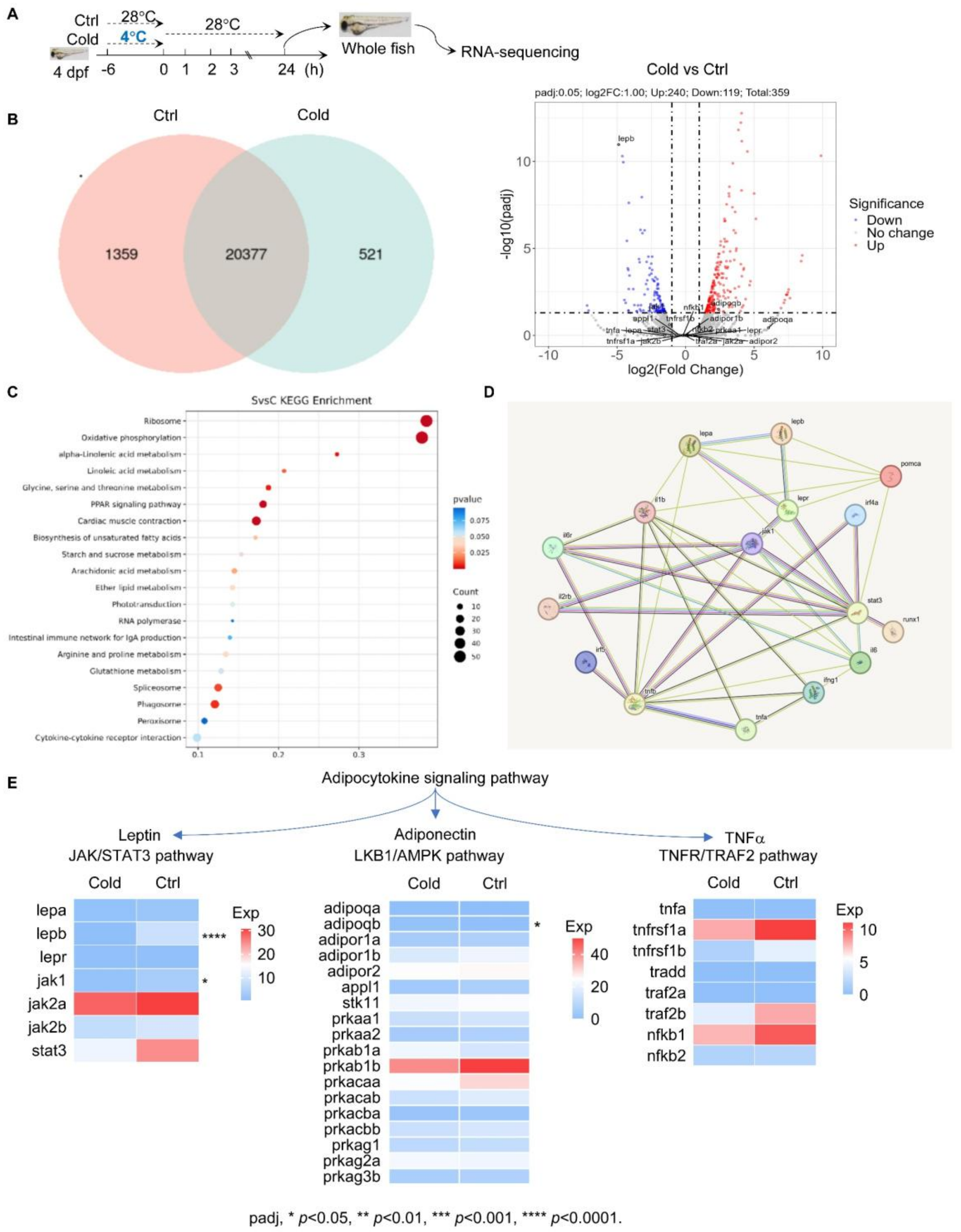

Figure S2. RNA-seq analysis in zebrafish following a single cold stress event reveals enrichment of metabolic-immune pathways and constructs a leptin-associated interaction network. (A) Experimental timeline of a single cold stress (4°C for 6 h) and RNA sequencing in 4 dpf zebrafish. **(B)** Analysis of differentially expressed genes (DEGs) between the cold stress and control groups. Left: Venn diagram showing the number of unique or shared differentially expressed genes among groups (fpkm>1).

Right: Volcano plot of fold changes values for all genes. **(C)** Bubble plot of KEGG pathway enrichment analysis for the DEGs. The y-axis shows the names of enriched pathways, the x-axis shows the enrichment factor, and the bubble size represents the number of DEGs. **(D)** A hierarchical view of the protein-protein interaction network constructed from the DEGs. Nodes represent proteins, and edges represent interaction relationships. Leptin is positioned as the top-level starting point in the network. **(E)** Heatmap analysis of differential gene expression related to key molecules in the three adipokine signaling pathways based on RNA-seq results.

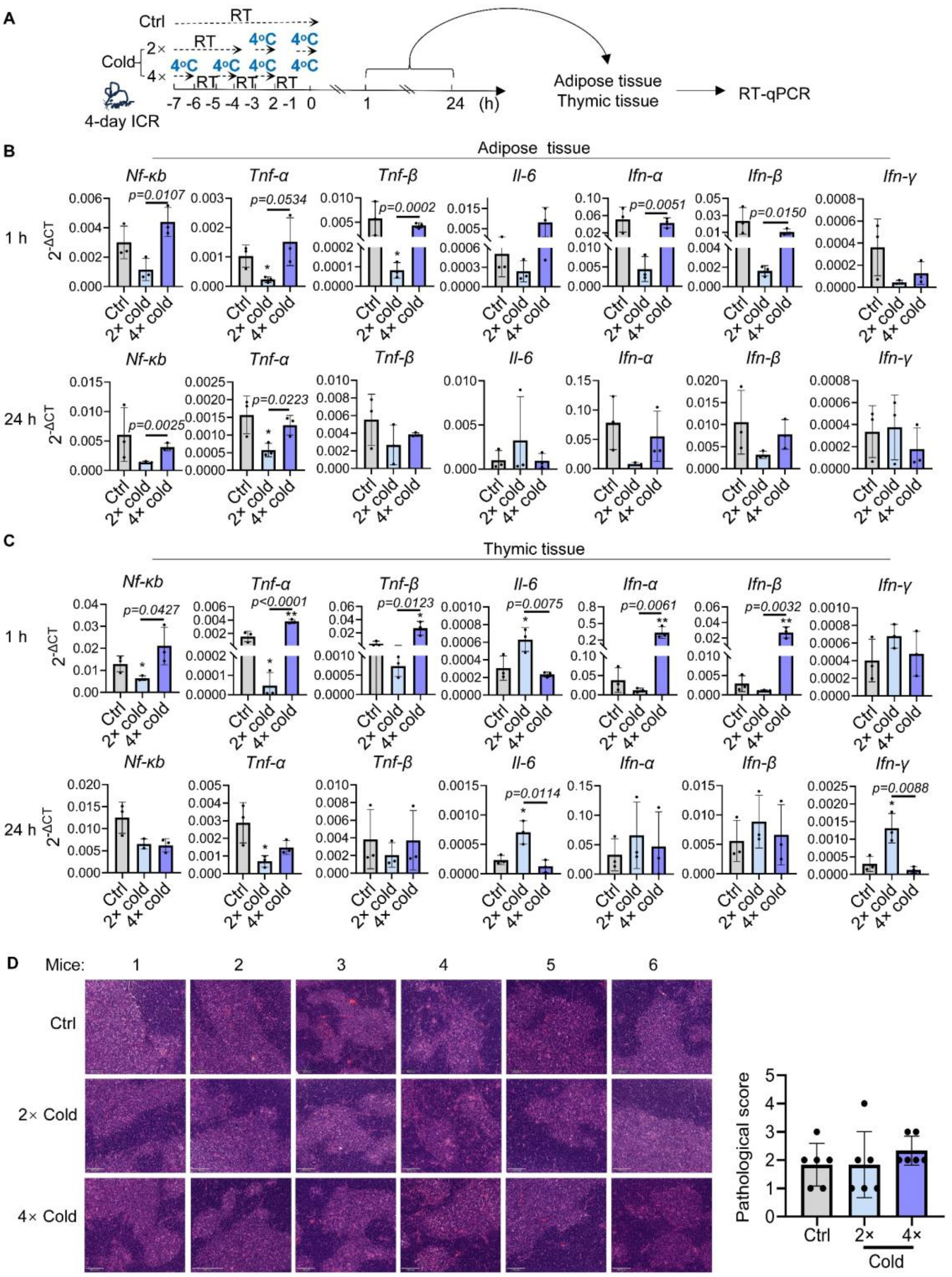

Figure S3. Effects of cyclic cold stress on the expression of inflammatory cytokines in neonatal mouse adipose and thymic tissues. **(A)** Experimental timeline. **(B)** mRNA levels of inflammatory cytokines in adipose tissue. **(C)** mRNA levels of inflammatory cytokines in thymic tissue. **(D)** Representative H&E-stained thymus tissue sections and corresponding thymus pathology scores for each group on 24 h post-cold stress. Each data point represents one mouse. All the experiments were repeated

three times. Data represent mean ± SD. * $p< 0.05$, **, $p <0.01$, ***, $p <0.001$, ****, $p <0.0001$.

Methods

Zebrafish larvae survivals under cold stress

Zebrafish larvae at 4 dpf and 7 dpf were subjected to continuous 4°C exposure for various time durations, followed by recovery at 28°C, and survival was monitored.

RNA sequencing

The RNA sequencing was completed by Zhongdi Huanyu (Xi'an, China) Biotechnology Co., Ltd., utilizing the Illumina second-generation high-throughput sequencing platform with the PE150 sequencing strategy. Total RNA was extracted using Trizol reagent (Invitrogen, USA) and treated with DNase I (Takara, Japan) to remove genomic DNA. RNA integrity and purity were assessed by 1% agarose gel electrophoresis, Agilent 2100 Bioanalyzer (Agilent Technologies, USA), and NanoDrop spectrophotometer (Thermo Scientific, USA). Sequencing libraries were constructed with the NEBNext® Ultra™ RNA Library Prep Kit for Illumina® (NEB, USA) using 1.5 μg of total RNA as input. The procedure included mRNA enrichment, fragmentation, cDNA synthesis, end repair, adapter ligation, size selection (200-250 bp), and PCR amplification. The final libraries were sequenced on an Illumina Novaseq 6000 platform to generate 150 bp paired-end reads.

Raw sequencing data were processed to remove adapters and low-quality reads, yielding high-quality clean reads. These clean reads were then aligned to the reference genome using STAR/HISAT2. Gene expression levels were quantified with HTSeq and normalized as FPKM (Fragments Per Kilobase of transcript per Million mapped fragments). Differential expression analysis was performed using DESeq2 (for samples with biological replicates) or edgeR (for samples without biological replicates), with significantly differentially expressed genes (DEGs) defined as those with an adjusted p-value < 0.05 and $|\log2(\text{fold change})| \geq 1$. Gene Ontology (GO) and Kyoto

Encyclopedia of Genes and Genomes (KEGG) pathway enrichment analyses of the DEGs were conducted using the GOseq and clusterProfiler R packages, respectively. A preliminary protein-protein interaction (PPI) network focused on leptin signaling and its associated molecules (Stat3, IRF4, IRF5) was constructed using the STRING database and visualized with Cytoscape.

RT-qPCR

mRNA was reverse transcribed into cDNA using a cDNA Synthesis Kit. qPCR was performed on the StepOne™ Real-Time PCR System (Applied Biosystems, Foster City, CA, USA) using a three-step SYBR Green fluorescence method with gene-specific primers (**Table S1**). The reaction conditions were as follows: 94°C for 5 s (1 cycle); followed by 40 cycles of 94°C for 5 s, 60°C for 30 s, and 72°C for 15 s. Target mRNA expression levels were normalized to *β-Actin* or *DADPH* mRNA and analyzed using the $2^{-\Delta Ct}$ method. Each qPCR plate included a no-template control.

Table S1. qPCR primers for target gene amplification from zebrafish and mice.

Species	Genes	Forward primer (5'-3')	Reverse primer (5'-3')
Mice	*Nf-κb*	GCATTCTGACCTTGCCTAT	CCAGTCTCCGAGTGAAGC
	Tnf-α	GTGCCTATGTCTCAGCCTCTT	GCCATAGAACTGATGAGAGGGA
	Tnf-b	GGTTCTCCACATGACACTGCT	AGAAGCGGACACCAGAGAGT
	Il-6	ACAAGTCCGGAGAGGAGACT	TTCTGCAAGTGCATCATCGT
	Ifn-α	TGTCTGATGCAGCAGGTGG	AAGACAGGGCTCTCCAGAC
	Ifn-b	TTACACTGCCTTTGCCATCCA	TGGAGAGCAGTTGAGGACATC
	Ifn-g	CGCTACACACTGCATCTTGG	CCATCCTTTTGCCAGTTCCTC

Thymus Pathological Analysis in Neonatal Mice

Thymus tissues were collected from 4-day-old neonatal mice 24 hours after exposure to 2× or 4× cold stress for pathological analysis. Thymus tissue sections were prepared and subjected to Hematoxylin and Eosin (HE) staining using the same protocol as for the lung tissues. Compared with the control group, the pathological changes of the thymus are scored on a 5-point scale, evaluating four criteria: thymus size, clarity of the corticomedullary junction, increased cellular density in the cortical/medullary areas, and the presence of hemorrhage.
